# Enhanced predictive performance of artificial intelligence in individualized ovarian stimulation of in vitro fertilization: a retrospective cohort study

**DOI:** 10.1186/s12916-026-04769-0

**Published:** 2026-03-10

**Authors:** Guiquan Wang, Minyue Tang, Liming Zhou, Fengcheng Li, Xiaoling Hu, Yunxian Yu, Haochao Ying, Ian Chew, Kai Zhu, Yimin Zhu

**Affiliations:** 1 Department of Reproductive Endocrinology, School of Medicine, Women’s Hospital, Zhejiang University, No. 1 Xueshi Road, Hangzhou, Zhejiang Province China; 2 Key Laboratory of Reproductive Genetics (Ministry of Education) and Women’s Reproductive Health Laboratory of Zhejiang Province, School of Medicine, Women’s Hospital, Zhejiang University, No. 1 Xueshi Road, Hangzhou, Zhejiang Province China; 3 Department of Reproductive Medicine, Women and Children’s Hospital, School of Medicine, Xiamen University, No. 10 Zhenhai Road, Xiamen, Fujian Province China; 4 Center for Reproductive Medicine, Ningbo Women & Children’s Hospital, Ningbo, Zhejiang Province China; 5 School of Pharmaceutical Sciences, Zhejiang University, Hangzhou, Zhejiang Province China; 6 Department of Epidemiology and Health Statistics, School of Public Health, Zhejiang University, Hangzhou, Zhejiang Province China; 7 Department of Big Data in Health Science, School of Public Health, Zhejiang University, Hangzhou, Zhejiang Province China; 8 Key Laboratory of Intelligent Preventive Medicine of Zhejiang Province, Hangzhou, Zhejiang Province China; 9 School of Medicine, Zhejiang University, Hangzhou, Zhejiang Province China; 10 Xiamen Key Laboratory of Reproduction and Genetics, Xiamen, Fujian Province China

**Keywords:** Artificial intelligence, Controlled ovarian stimulation, In vitro fertilization, Low ovarian response, Hyper ovarian response, Machine learning, Personalized medicine

## Abstract

**Background:**

Over 2.5 million cycles in vitro fertilization (IVF) are conducted annually, and numbers are expected to rise with the aging population. The controlled ovarian stimulation (COS) process, key to IVF success, is inherently complex. Given advances in artificial intelligence (AI), this study investigated whether a series of AI models can outperform traditional clinical practices in predictive accuracy and COS optimization.

**Methods:**

This retrospective cohort study analyzed first-cycle ovarian stimulation patients (Oct 2017–Dec 2020) and was validated using an independent cohort (Jan 2018–Jan 2022). Six AI algorithms and 73 variables were screened. A four-submodel strategy included risk prediction models for low and hyper ovarian response (LORRM, HORRM) and strategy deployment models (LORSM, HORSM) for managing critical COS components. Feature importance was assessed using Shapley additive explanations, with sensitivity analyses performed for robustness. The ability to propose effective COS strategies was also retrospectively assessed.

**Results:**

A four-submodel system prototype using extreme gradient boosting trees was developed. All submodels showed superior discrimination compared to conventional ovarian reserve markers (AUC, 95% CI: LORSM, 0.95 [0.94–0.96]; LORRM, 0.93 [0.92–0.94]; HORSM, 0.90 [0.88–0.91]; HORRM, 0.89 [0.87–0.91]. DeLong *P* < 0.001 for all). They demonstrated adequate calibration (Brier scores of four submodels ranged from 0.064 to 0.072), promising performance in external validation (AUCs ranging from 0.84 to 0.88) and sensitivity analyses. Among COS components, COS protocol and recombinant follicle-stimulating hormone (FSH) use had the largest impact on low and hyper response risks, respectively, with FSH starting dose ranking third. Diastolic blood pressure, alanine aminotransferase, and white blood cell count predicted low response, while basal luteinizing hormone (LH) levels and platelet count were key for hyper response. Several were newly identified potential biomarkers. LORSM and HORSM identified effective strategies with precision of 95.5% (95% CI, 94.6–96.4%) and 98.4% (95% CI, 98.0–98.9%), respectively.

**Conclusions:**

The AI-based system demonstrated superior detection of abnormal ovarian responses and effective individualized COS design compared to conventional clinical practice while maintaining transparency. The system identified potential biomarkers beyond conventional ovarian reserve markers and offered new insights for optimizing IVF, showing promise for advancing personalized reproductive medicine.

**Supplementary Information:**

The online version contains supplementary material available at 10.1186/s12916-026-04769-0.

## Background

In vitro fertilization (IVF) has contributed to more than 8 million births, and over 2.5 million cycles are being conducted annually and the number is expected to continue increasing [1–3]. The unpredictability of ovarian response can result in failure of controlled ovarian stimulation (COS), high risks of complications, and economic burden [4]. It is far from easy to achieve personalization of ovarian stimulation, of which the difficulties mainly derive from the individual variability, long duration of the treatment, vast number of drugs, and pretreatment processes. Low ovarian response (LOR) affects an estimated 6–35% of women and cause excessive gonadotropin consumption, repeated IVF cycles, and reduced pregnancy chances [5]. Hyper ovarian response (HOR) is associated with lower quality of oocyte/embryos, decreased endometrial receptivity [6], and an elevated risk of life-threatening ovarian hyperstimulation syndrome [7].

Improved prediction offers important clinical benefits beyond current practice. For patients at risk of low response, accurate assessment enables informed counseling and shared decision-making, potentially reducing treatment dropout. For those at risk of hyper response, early identification permits pre-emptive protocol optimization to avoid OHSS and costly freeze-all cycles. Many years of research have aimed at optimizing this specific phase. Current practice is usually based on empirical combinations of traditional ovarian reserve markers (ORMs), including age, anti-Müllerian hormone (AMH), basal antral follicle count (AFC), and follicle-stimulating hormone (FSH) [8], while little is known for each marker’s precise weight [9]. Previous studies have proposed models to predict the risks of LOR or HOR using ORMs in specific scenarios or populations [10–14]. Great attention has also been put on optimizing the gonadotropin dosing to achieve an expected normal ovarian response [15, 16], although this may introduce overestimation and deepen the prejudice of “the more oocytes the better” [17]. In fact, there are far more potential risk factors and COS components that may affect ovarian response [18–21]. However, as the number of variables and underlying interactions significant increases substantially, classical statistical techniques could be limited by its strict assumptions and computational intractability [22, 23]. Therefore, we hypothesized that artificial intelligence (AI) could improve prediction by effectively extracting meaningful patterns and attributing relevance especially from large-scale and multidimensional data. On the other hand, given the impending artificial intelligence (AI) explosion and human embrace of AI, it is valuable to ascertain whether AI, under the premise of transparency and comprehensive evaluations, do outperform the conventional clinical practice in predictive accuracy and can automate the design of COS treatment for clinical utility [24].

In this study, we developed a series of AI models and validated them using an independent, retrospectively collected external cohort. These models could serve as the foundation for a future AI-based clinical decision support system. These models both predict the risks of abnormal ovarian responses and propose individualized COS strategies. Evaluating six representative AI algorithms, a four-submodel strategy was employed to construct the system which integrated risk prediction submodels for LOR and HOR, along with corresponding submodels for scheduling COS strategies. To make models transparent, the contribution of key predictors to the predictions was calculated. Superiority analyses were conducted to explore whether the models outperformed conventional ORMs. The submodels were further validated in an independent cohort and tested under various constrained conditions. We also retrospectively assessed the effectiveness of the strategy submodels in proposing individualized COS strategies.

## Methods

### Study population

To ensure the robustness and generalizability of our models, we used two distinct cohorts from different tertiary care hospitals. The derivation cohort (*n* = 12,780) was from Zhejiang University School of Medicine Women’s Hospital, with data collected between October 2017 and December 2020. The external validation cohort (*n* = 6323) was from Ningbo Women & Children’s Hospital, with data collected between January 2018 and January 2022. The intentional difference and overlap in data collection periods were designed to test the model’s temporal robustness against potential shifts in clinical practices or patient populations over time. The study population in both the derivation and validation cohorts consisted exclusively of women of Asian ethnicity. To ensure the model is applicable to a wide range of clinical scenarios, eligibility criteria were intentionally broad to include a population with a wide spectrum of ages, causes of infertility, and baseline ovarian reserve markers. We only included the first COS cycles to avoid variations in treatment strategies and potential changes in patients’ physical and mental status due to previous cycles not resulting in live births. To better represent the general population, eligible criteria were set at a broad level (Additional file 1: Fig. S1). Patients with chromosomal abnormalities were excluded. Cycles with incomplete COS data or implausible values were excluded. This paper followed per the Transparent Reporting of a Multivariable Prediction Model for Individual Prognosis or Diagnosis guideline and Prediction Model Risk of Bias Assessment Tool reporting guideline [25, 26].

### Outcome measures

We classify the number of retrieved oocytes and treat LOR and HOR as separate prediction tasks instead of directly predicting the number of retrieved oocytes as a continuous variable. Initial exploratory analyses using regression models achieved an RMSE of 3.5 oocytes, which is clinically unacceptable for identifying low responders (e.g., a prediction of 4 oocytes would have a 95% interval spanning 0–11). This approach is based on the facts that LOR and HOR have fundamental differences in terms of incidence, etiology, and pathophysiological mechanisms. It is possible that they have different predictors or that the same predictors contribute differently in terms of magnitude and direction to the predictions. This binary classification approach outperformed both continuous prediction and three-class classification in preliminary analyses and aligns with clinical decision-making where each risk is evaluated independently.

We do not directly predict LOR according to the Bologna or Poseidon criteria because age, AMH, and AFC are already known before initiating ovarian stimulation [27, 28]. This definition is reasonable for diagnosis purposes. However, for prediction tasks, we prefer to use these three ORMs as predictors along with other factors to predict the unknown ovarian response. To keep in accordance with the Bologna and Poseidon criteria, LOR was categorized as the retrieval of less than 4 oocytes. Considering no uniform criterion for HOR, we defined it as retrieval of more than 20 oocytes which is deemed associated with both a significant risk increase in ovarian hyperstimulation syndrome and a steady decline of live birth rate [16, 29, 30].

### Candidate clinical covariates

A total of 73 variables of the derivation cohort were prescreened, which covered detailed information of demographic, anthropometric, medical history, laboratory parameters, and COS treatments (Additional file 2: Table S1). These variables are routinely collected as part of mandatory pretreatment screening in our clinical setting and impose no additional patient burden. Four critical COS component variables (COS protocol, FSH starting dose, use of recombinant FSH, and luteinizing hormone [LH] supplementation) were categorized into 7, 6, 3, and 3 classes, respectively. In this study, deploying a COS strategy was defined as scheduling a plausible combination of the four COS components. Descriptions of the standard COS process, categorization, measurements of AFC and hormones, and diagnostic criteria for the reproductive disorders in this study were provided in Additional file 3: Supplementary methods [31, 32]. After exclusion of 11 covariates for > 15% missingness, 6 for large imbalance, and 1 for large correlation (coefficient > 0.7), 55 candidate variables were used in subsequent modeling processes.

### Design of the AI-based system prototype

We employed a four-submodel strategy to accomplish four separate predictive tasks, specifically, developing two risk prediction models that integrate baseline features to predict LOR and HOR (i.e., LORRM and HORRM), and developing two strategy deployment models that incorporate baseline features along with four schedulable COS components to mitigate the risks of LOR and HOR (i.e., LORSM and HORSM). Risk submodels will directly provide the predicted classification and risks of abnormal ovarian responses. Strategy submodels will (1) exhaust all clinically plausible combinations of four COS components, (2) filter those predicted as non-LOR/non-HOR, (3) sort them in descending order based on the predicted risks, and (4) propose the optimal list of top *N* effective COS strategies. In cases where both models simultaneously predict positive (i.e., a patient is flagged as high risk for both LOR and HOR), the system displays a conflict alert with both predicted probabilities, highlights the classification with the higher probability as the primary risk, and flags the case for additional clinical review. The strategy submodels evaluate 83 predefined combinations of COS components and rank them by predicted risk, functioning as simulation tools rather than optimizers (detailed workflow in Additional file 3: Supplementary methods) [33–36].

### Model development and interpretation

**Fig. 1:**
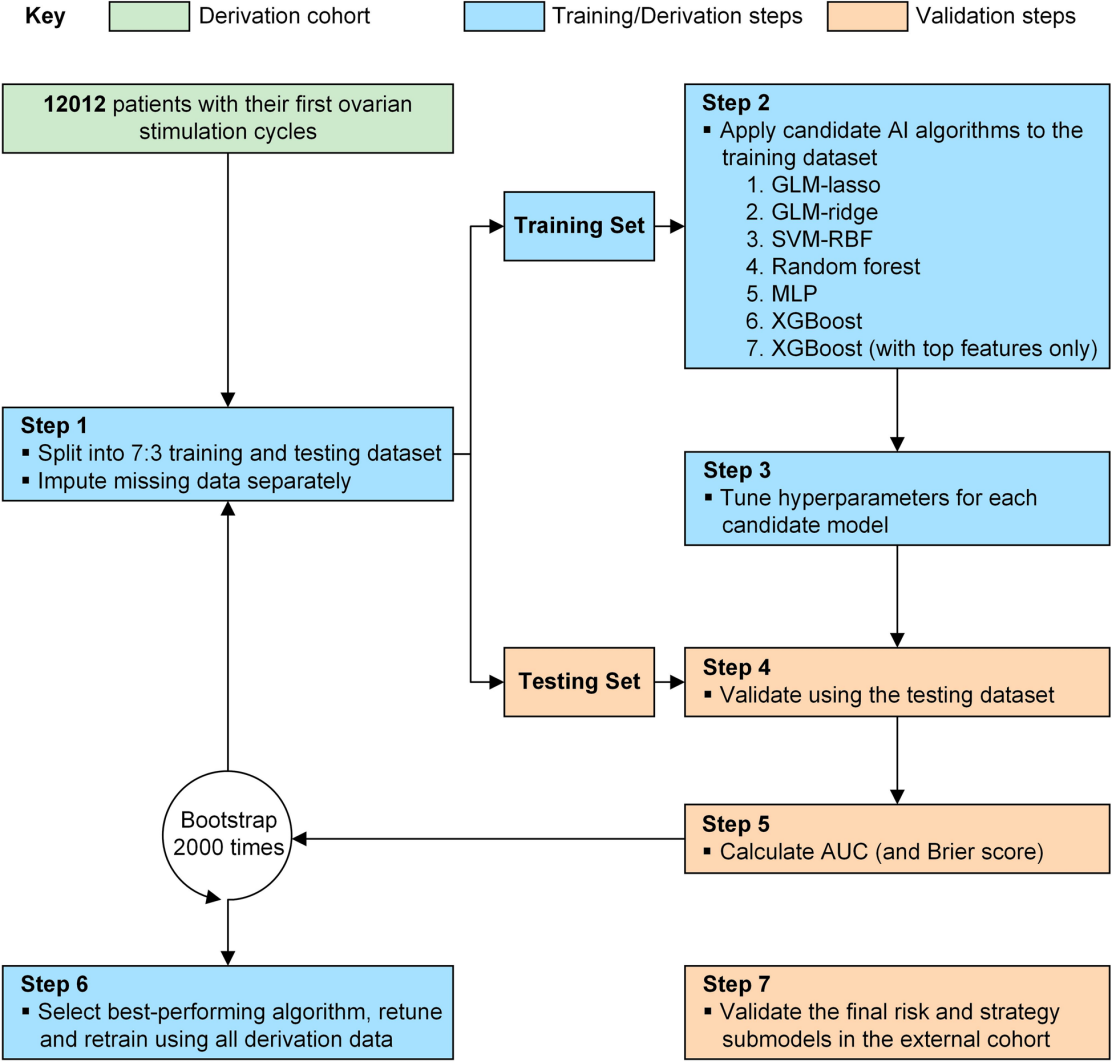
Analysis overview for identifying the best-performing tool to develop the AI-based system prototype. Abbreviations: AUC, areas under the receiver operating characteristic curve; GLM, generalized linear model; AI, artificial intelligence; MLP, multilayer perceptron; SVM-RBF, support vector machine with radial basis functions; XGBoost, extreme gradient boosting trees

The overview of model development and validation was displayed in Fig. 1. Missing data were imputed separately using multivariate imputation by chained equations with random forests [37]. A strategy-model-determined scheme was used to reduce the complexity of the modeling process. That was, the top important risk factors and the algorithm for developing four submodels were determined by the outperforming full-variable strategy models. Six representative AI algorithms were evaluated: (1) LASSO logistic regression, (2) ridge logistic regression, (3) support vector machine with radial basis functions, (4) random forest, (5) multilayer perceptron, and (6) extreme gradient boosting trees (XGBoost). A Bayesian optimization method was performed to tune hyperparameters before each modeling process on basis of stratified five-fold cross-validation [34]. All splits were stratified by ovarian response status. Hyperparameter search spaces and optimized values are detailed in Additional file 2: Table S4. A methodology of Shapley additive explanations (SHAP) was used to obtain feature importance, select top important features, and interpretate predictions [35, 36, 38]. XGBoost and SHAP are inherently robust to moderate multicollinearity among predictors. Detailed descriptions of the AI algorithms, feature selection process, and global and local interpretations were provided in Additional file 3: Supplementary methods. To assess potential multicollinearity among predictors, we computed a Pearson correlation matrix for all top features identified by SHAP analysis.

### Model evaluations

All submodels included in the AI-based system were both internally and externally validated. To confirm no significant decrease of performance due to reduction of integrated features and no significant information bias introduced by imputation, full-variable models and models using only complete case data were both developed for comparison.

For a superiority analysis (40 comparisons in total), the submodels were submitted to compare with the models that only incorporated the conventional ORMs (for comparing strategy submodels, the COS component variables were added) using the similar modeling approach. Several sensitivity analyses were performed to further assess the performance of the submodels under constrained conditions, such as missing certain ORMs and putting them in prespecified populations (16 tests in total). Details of the design were described in Additional file 3: Supplementary methods. To retrospectively assess the efficacy of strategy submodels proposing COS strategies, the actual strategies of non-LOR/non-HOR patients were considered as effective strategies, and those of LOR/ HOR patients as ineffective ones. Then what we focused could be divided into three aspects: (1) the overall ability of identifying effective COS strategies (actual non-LOR/non-HOR strategies), assessed by balanced accuracy and Matthews correlation coefficient [39–41]; (2) the probability of effective strategies appearing in the proposed strategy list (predicted non-LOR/non-HOR strategies), assessed by precision for negative class; (3) the ability to avoid ineffective COS strategies (actual LOR/HOR strategies) appearing in the proposed list or to successfully detect them, assessed by true positive rate.

### Statistical analysis

Model discrimination was evaluated by receiver operating characteristic curve analysis providing area under the curve (AUC) with 95% confidence intervals (95% CI, bootstrapping 2000 replicates). Maximizing the Youden index was used to obtain the optimal cutoffs discriminating LOR (or HOR) from non-LOR (or non-HOR) [42]. To assess differences between AUCs, DeLong test was performed with a two-tailed *P* < 0.05 indicating statistical significance [43]. Model calibration was assessed by Brier scores. Five metrics for assessing the ability of proposing effective COS strategies are defined as

$$Precision \left(negative class\right)=\frac{TN}{TN+FN},$$

$$True Positive Rate=\frac{TP}{TP+FN},$$

$$True Negative Rate=\frac{TN}{TN+FP},$$

$$Balanced Accuracy=\frac{1}{2}\left(\frac{TP}{TP+FN}+\frac{TN}{TN+FP}\right),$$

$$MCC=\frac{TP\times TN-FP\times FN}{\sqrt{(TP+FP)(TP+FN)(TN+FP)(TN+FN)}}$$

where *TP* and *TN* indicate true positives and negatives, and *FP* and *FN* indicate false positives and negatives, respectively. A Matthews correlation coefficient of 1 indicates perfect prediction, 0 indicates no better than random guessing, and − 1 indicates complete disagreement with the actual values. All data manipulation, modeling processes, and statistical analyses were performed in R version 4.0.5 (R Foundation, Austria). A full list of tools used in this study is provided in Additional file 2: Table S2.

## Results

### Study population characteristics

The main characteristics of the derivation and external validation cohorts, stratified by ovarian response groups, are summarized in Table 1. After exclusions, the derivation cohort included 12,012 patients, distributed as follows: 1714 (14.3%) classified as having LOR (median [IQR] age: 35 [32–39] years), 9175 (76.4%) with a normal ovarian response (age: 32 [29–34] years), and 1123 (9.3%) classified as HOR (age: 29 [26–31] years). The external validation cohort consisted of 5702 patients, with 882 (15.5%) LOR, 4404 (77.2%) normal response, and 416 (7.3%) HOR, reflecting a distribution similar to that of the derivation cohort. Patients with LOR were generally older, had lower AMH levels, elevated FSH levels, and reduced AFC in both cohorts, indicating a diminished ovarian reserve. Additionally, LOR patients had higher diastolic blood pressure and lower white blood cell counts. Detailed sample sizes used for various modeling processes and analyses are presented in Additional file 1: Fig. S1.

**Table 1:** Main characteristics of the derivation and external validation cohorts across ovarian response groups

Main characteristics	Derivation cohort			External validation cohort		
	LOR (*n* = 1714)	Normal (*n* = 9175)	HOR (*n* = 1123)	LOR (*n* = 882)	Normal (*n* = 4404)	HOR (*n* = 416)
Age, y	35 (32–39)	32 (29–34)	29 (26–31)	34 (30–37)	31 (29–33)	29 (27–31)
Duration of infertility, y	3 (2–5)	3 (2–4)	2 (1–3)	3 (2–5)	3 (2–4)	2 (1–3)
Weight, kg	55 (50–60)	55 (50–60)	55 (50–60)	55 (50–60)	55 (50–61)	55 (51–62)
Diastolic blood pressure, mmHg	71 (66–78)	70 (66–77)	70 (66–78)	70 (64–76)	70 (64–76)	70 (63–77)
Basal follicle-stimulating hormone, IU/L	9.3 (7.3–12.1)	7.4 (6.1–8.9)	6.4 (5.4–7.8)	9.2 (7.1–11.8)	7.3 (6.0–8.8)	6.4 (5.3–7.8)
Basal luteinizing hormone, IU/L	4.6 (3.3–6.3)	4.9 (3.6–6.4)	5.8 (4.3–8.5)	3.7 (2.5–5.3)	3.9 (2.6–5.6)	4.4 (2.8–6.6)
Basal progesterone, ng/mL	0.6 (0.4–0.8)	0.6 (0.4–0.8)	0.6 (0.4–0.8)	0.5 (0.4–0.7)	0.6 (0.4–0.8)	0.6 (0.4–0.8)
White blood cell count, 10^9^/L	6.2 (5.2–7.5)	6.6 (5.4–8.1)	6.7 (5.6–8.3)	7.0 (5.7–8.4)	7.3 (6.0–8.9)	7.5 (6.1–9.0)
Red blood cell count, 10^12^/L	4.4 (4.1–4.6)	4.4 (4.2–4.6)	4.4 (4.2–4.6)	4.4 (4.2–4.6)	4.4 (4.2–4.6)	4.4 (4.2–4.6)
Platelet, 10^9^/L	234 (198–275)	233 (198–273)	233 (199–269)	245 (210–283)	247 (210–287)	244 (208–289)
Alanine aminotransferase, U/L	15 (12–21)	15 (11–20)	15 (12–21)	13 (10–18)	13 (10–19)	14 (10–20)
Anti-Müllerian hormone, ng/mL	0.8 (0.4–1.4)	3.0 (1.8–4.6)	5.9 (4.4–8.7)	1.3 (0.7–2.0)	3.4 (2.1–5.6)	7.2 (4.6–10.9)
Basal antral follicle count, *n*	4 (3–7)	10 (7–15)	15 (12–20)	5 (3–7)	10 (7–15)	17 (12–20)
Tubal factor, *n* (%)						
No	1058 (61.7)	5120 (55.8)	661 (58.9)	527 (59.8)	2374 (53.9)	236 (56.7)
Yes	656 (38.3)	4055 (44.2)	462 (41.1)	355 (40.2)	2030 (46.1)	180 (43.3)
Endometriosis, *n* (%)						
No	1088 (63.5)	6312 (68.8)	6312 (85.2)	584 (66.2)	3078 (69.9)	369 (88.8)
Yes	626 (36.5)	2863 (31.2)	2863 (14.8)	298 (33.8)	1326 (30.1)	47 (11.3)
Polycystic ovary syndrome, *n* (%)						
No	1685 (98.3)	8487 (92.5)	799 (71.1)	851 (96.5)	3794 (86.1)	280 (67.3)
Yes	29 (1.7)	688 (7.5)	324 (28.9)	31 (3.5)	610 (13.9)	136 (32.7)
Primary ovarian insufficiency or diminished ovarian reserve, *n* (%)						
No	459 (26.8)	7982 (87.0)	1118 (99.6)	429 (48.6)	4031 (91.5)	416 (100.0)
Yes	1255 (73.2)	1193 (13.0)	5 (0.4)	453 (51.4)	373 (8.5)	0 (0.0)
Ovarian stimulation protocol^a^, *n* (%)						
Long GnRH agonist	96 (5.6)	3426 (37.3)	550 (49.0)	222 (25.2)	3169 (72.0)	353 (84.9)
Ultra-long GnRH agonist	39 (2.3)	377 (4.1)	22 (2.0)	57 (6.5)	548 (12.4)	42 (10.1)
Short GnRH agonist	142 (8.3)	490 (5.3)	11 (1.0)	0 (0)	0 (0)	0 (0)
GnRH antagonist	540 (31.5)	4204 (45.8)	489 (43.5)	392 (44.4)	616 (14.0)	20 (4.8)
Progestin-primed ovarian stimulation	203 (11.8)	235 (2.6)	28 (2.5)	102 (11.6)	35 (0.8)	1 (0.2)
Mild or Natural	423 (24.7)	142 (1.5)	1 (0.1)	83 (9.4)	14 (0.3)	0 (0)
Other^b^	271 (15.8)	301 (3.3)	22 (2.0)	26 (2.9)	22 (0.5)	0 (0)
Follicle-stimulating hormone starting dose^a^, *n* (%)						
≤ 100 IU	13 (0.8)	275 (3.0)	83 (7.4)	2 (0.2)	9 (0.2)	0 (0)
150 IU	147 (8.6)	3251 (35.4)	634 (56.5)	79 (9.0)	808 (18.3)	97 (23.3)
200 IU	37 (2.1)	763 (8.3)	138 (12.3)	19 (2.2)	187 (4.2)	36 (8.7)
225 IU	510 (29.7)	3530 (38.5)	185 (16.5)	476 (54.0)	2639 (59.9)	236 (56.7)
≥ 300 IU	111 (6.5)	678 (7.4)	32 (2.8)	95 (10.8)	690 (15.7)	46 (11.1)
Non-GnRH^c^	896 (52.3)	678 (7.4)	51 (4.5)	211 (23.9)	71 (1.6)	1 (0.2)
Using rFSH or uFSH^a^						
uFSH	567 (33.1)	2963 (32.3)	136 (12.1)	671 (76.1)	4333 (98.4)	415 (99.8)
rFSH	252 (14.7)	5534 (60.3)	936 (83.3)	0 (0)	0 (0)	0 (0)
Non-GnRH^c^	895 (52.2)	678 (7.4)	51 (4.6)	211 (23.9)	71 (1.6)	1 (0.2)
Luteinizing hormone supplementation^a^, *n* (%)						
No	273 (15.9)	3283 (35.8)	489 (43.5)	348 (39.5)	1462 (33.2)	146 (35.1)
Yes	545 (31.8)	5214 (56.8)	583 (51.9)	323 (36.6)	2871 (65.2)	269 (64.7)
Non-GnRH^c^	896 (52.3)	678 (7.4)	51 (4.6)	211 (23.9)	71 (1.6)	1 (0.2)
No. of oocytes retrieved	2 (1–3)	10 (7–14)	25 (22–28)	2 (1–3)	9 (7–13)	23 (21–25)

Values presented are before imputation. Categorical and continuous variables are reported as no. (%) and median (interquartile range), respectively

Abbreviations: *AFC*, antral follicle count; *ALT*, alanine aminotransferase; *AMH*, anti-Müllerian hormone; *DBP*, diastolic blood pressure; *DOR*, diminished ovarian reserve; *FSH*, follicle-stimulating hormone; *GnRH*, gonadotropin-releasing hormone; *LH*, luteinizing hormone; *PCOS*, polycystic ovarian syndrome; *POI*, primary ovarian insufficiency; *PPOS*, progestin-primed ovarian stimulation protocol; *rFSH*, recombinant follicle-stimulating hormone; *uFSH*, urinary follicle-stimulating hormone

^a^Four schedulable ovarian stimulation components

^b^Other protocols included using gonadotropin alone and using gonadotropin-releasing hormone alone

^c^Neither GnRH agonist nor antagonist protocols were used

### Derivation of the AI-based submodels

Among six representative AI algorithms, XGBoost demonstrated the highest performance in developing full-variable LOR and HOR strategy models, with AUCs of 0.95 (95% CI: 0.94–0.96) for LOR and 0.90 (95% CI: 0.89–0.91) for HOR, and corresponding Brier scores of 0.064 and 0.071, respectively (Additional file 1: Fig. S2). The application of multiple imputation did not introduce significant bias, as indicated in Additional file 2: Table S3. Based on the feature importance, 13 and 10 key predictors were selected to be incorporated in the final LOR and HOR submodels, respectively (Additional file 1: Fig. S3). Optimized hyperparameters throughout the model derivation processes were provided in Additional file 2: Table S4. Retrained using the entire derivation cohort, four XGBoost-based submodels were finally developed: LORRM, HORRM (risk prediction models) and LORSM, HORSM (strategy deployment models).

### The AI-based models outperformed conventional clinical practice

**Fig. 2:**
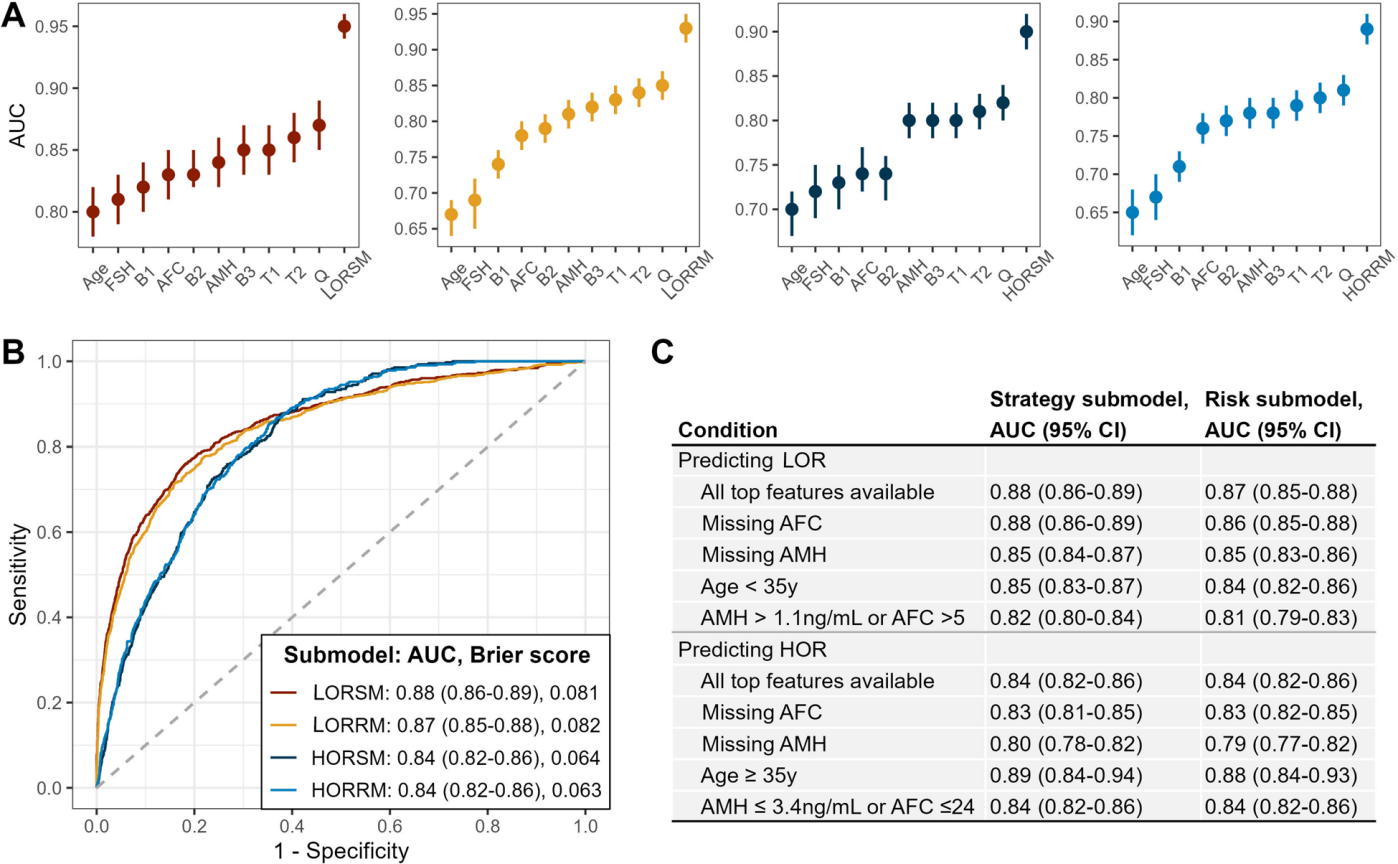
Contribution profiles of the most important features. **A** Alluvial plot of top important features included in the AI-based system identified by the variable importance metric (absolute mean SHAP values) in the derivation cohort. **B** SHAP summary plot of LORRM (risk prediction submodel for low ovarian response). **C** SHAP summary plot of HORRM (risk prediction submodel for hyper ovarian response). Abbreviations: AFC, antral follicle count; ALT, alanine aminotransferase; AMH, anti-Müllerian hormone; DBP, diastolic blood pressure; DOR, diminished ovarian reserve; FSH, follicle-stimulating hormone; LH, luteinizing hormone; OS, ovarian stimulation; PCOS, polycystic ovarian syndrome; POI, primary ovarian insufficiency; RBC, red blood cell count; rFSH, recombinant follicle-stimulating hormone; SHAP, Shapley additive explanations; uFSH, urinary follicle-stimulating hormone; WBC, white blood cell count

The results of performance comparisons were summarized in Table 2 and visually inspected in Fig. 2A.

**Table 2:** Model evaluations including the superiority analysis comparing the AI submodels with models integrating combinations of conventional ovarian reserve markers

Model	Predicting LOR		Predicting HOR	
	AUC (95% CI)	DeLong test, *P* value	AUC (95% CI)	DeLong test, *P* value
Strategy models				
Strategy submodels^a^	0.95 (0.94–0.96)	Reference	0.90 (0.88–0.91)	Reference
Full-variable strategy models	0.95 (0.94–0.96)	> 0.99	0.90 (0.89–0.92)	> 0.99
Strategy model trained using complete data^b^	0.94 (0.92–0.96)	0.50	0.90 (0.88–0.92)	0.85
Age	0.80 (0.78–0.82)	< 0.001	0.70 (0.67–0.72)	< 0.001
FSH	0.81 (0.79–0.83)	< 0.001	0.72 (0.69–0.75)	< 0.001
AFC	0.83 (0.81–0.85)	< 0.001	0.74 (0.72–0.77)	< 0.001
AMH	0.84 (0.82–0.86)	< 0.001	0.80 (0.78–0.82)	< 0.001
Age + FSH	0.82 (0.80–0.84)	< 0.001	0.73 (0.70–0.75)	< 0.001
Age + AFC	0.83 (0.82–0.85)	< 0.001	0.74 (0.71–0.76)	< 0.001
Age + AMH	0.85 (0.83–0.87)	< 0.001	0.80 (0.78–0.82)	< 0.001
Age + AMH + FSH	0.85 (0.83–0.87)	< 0.001	0.80 (0.78–0.82)	< 0.001
Age + AMH + AFC	0.86 (0.84–0.88)	< 0.001	0.81 (0.79–0.83)	< 0.001
Age + AMH + FSH + AFC	0.87 (0.85–0.89)	< 0.001	0.82 (0.80–0.84)	< 0.001
Risk prediction models				
Risk prediction submodels^a^	0.93 (0.91–0.95)	Reference	0.89 (0.87–0.91)	Reference
Full-variable risk prediction models	0.93 (0.91–0.95)	> 0.99	0.89 (0.87–0.91)	> 0.99
Risk prediction models trained using complete data^b^	0.92 (0.90–0.94)	0.49	0.89 (0.86–0.91)	0.90
Age	0.67 (0.64–0.69)	< 0.001	0.65 (0.62–0.68)	< 0.001
FSH	0.69 (0.65–0.72)	< 0.001	0.67 (0.64–0.70)	< 0.001
AFC	0.78 (0.76–0.80)	< 0.001	0.76 (0.74–0.78)	< 0.001
AMH	0.81 (0.79–0.83)	< 0.001	0.78 (0.76–0.80)	< 0.001
Age + FSH	0.74 (0.72–0.76)	< 0.001	0.71 (0.69–0.73)	< 0.001
Age + AFC	0.79 (0.77–0.81)	< 0.001	0.77 (0.75–0.79)	< 0.001
Age + AMH	0.82 (0.80–0.84)	< 0.001	0.78 (0.76–0.80)	< 0.001
Age + AMH + FSH	0.83 (0.81–0.85)	< 0.001	0.79 (0.77–0.81)	< 0.001
Age + AMH + AFC	0.84 (0.82–0.86)	< 0.001	0.80 (0.78–0.82)	< 0.001
Age + AMH + FSH + AFC	0.85 (0.83–0.87)	< 0.001	0.81 (0.79–0.83)	< 0.001

Risk prediction models for comparison only incorporated currently used ovarian reserve markers. Strategy models for comparison additionally incorporated four schedulable ovarian stimulation components

Abbreviations: *AFC*, antral follicle count; *AMH*, anti-Müllerian hormone; *AUC*, area under the receiver operating characteristic curve; *FSH*, follicle-stimulating hormone; *HOR*, hyper ovarian response; *LOR*, low ovarian response

^a^Submodels included in the AI-based system

^b^Models derived from the complete case data using a similar development approach as the submodels

#### The models based on conventional ovarian reserve markers

Models predicting LOR also using the XGBoost algorithm incorporating single or combined conventional ovarian reserve markers (ORM models) achieved a range of AUCs as 0.80 to 0.87, while the AUC range for predicting HOR is 0.70 to 0.82. Whether predicting LOR or HOR, the predictive power is strongest when incorporating all four markers (age, AMH, AFC, FSH).

#### The models beyond conventional indicators

All four AI-based submodels demonstrated significantly superior performance over their corresponding ORM models (AUCs [95% CIs]: LORSM, 0.95 [0.94–0.96]; LORRM, 0.93 [0.92–0.94]; HORSM, 0.90 [0.88–0.91]; HORRM, 0.89 [0.87–0.91]. DeLong *P* < 0.001 for all comparisons). These AI-based submodels also exhibited promising calibration performance (Brier scores: LORSM, 0.064; LORRM, 0.065; HORSM, 0.071; HORRM, 0.072). The inclusion of only top contributing variables in the submodels did not result in a significant decrease in performance compared to full-variable models (Delong *P* > 0.99 for all). The submodels demonstrated comparable performance to their corresponding models derived from complete case data, confirming no significant information bias introduced by the missingness imputation process (Delong *P* ≥ 0.49 for all).

Figure 2B shows the discrimination and calibration performance of four submodels in the external validation. As for sensitivity and subgroup analyses, the submodels present an acceptable decline of predictive ability in artificially predefined populations and in the absence of specific ORMs (Fig. 2C). By comparison, LOR submodels underwent the least AUC decline in absence of AFC but the largest decline in the population only including patients with good ovarian reserve. HOR submodels were most affected when AMH was missing.

### Global interpretability of the AI-based models

**Fig. 3:**
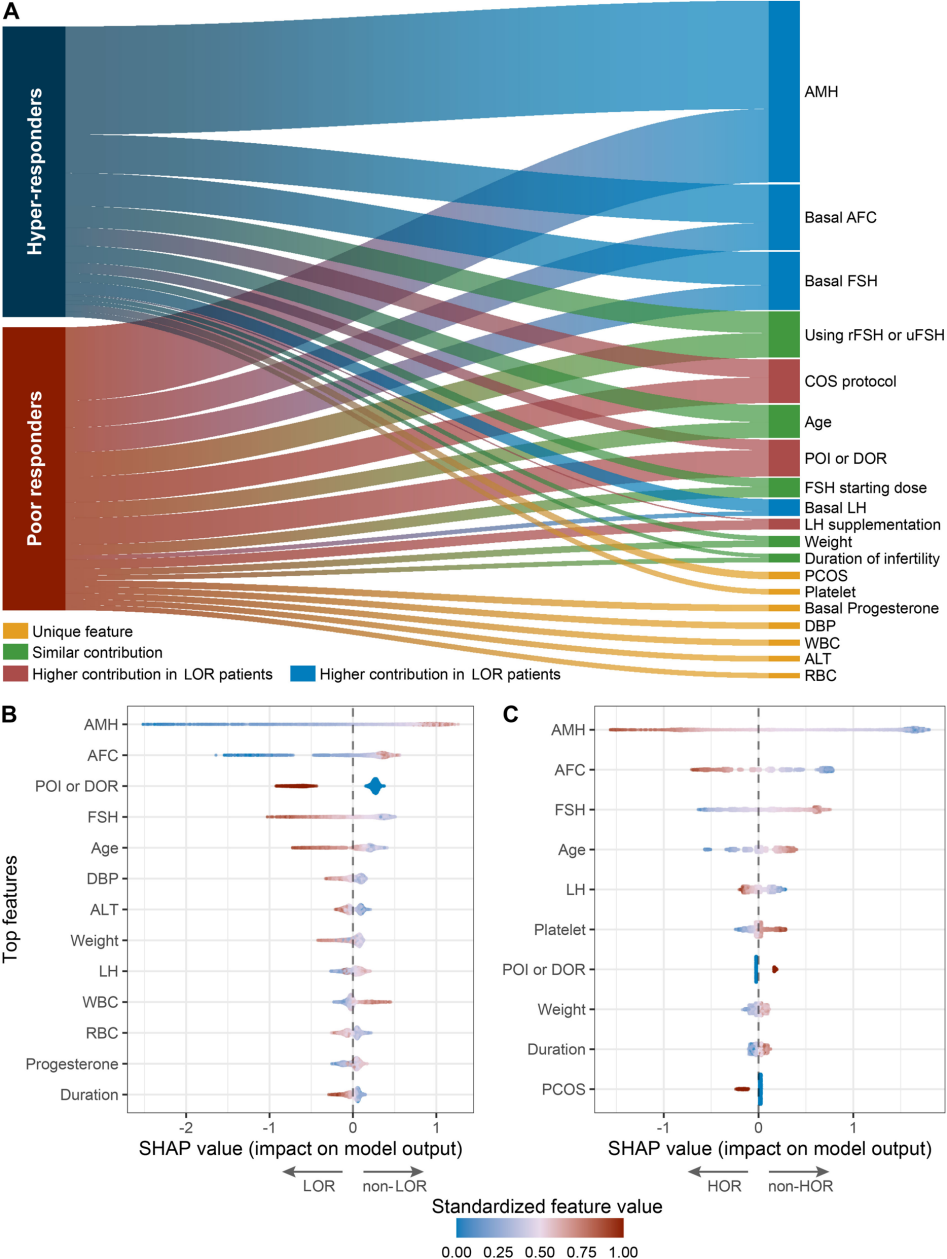
Results of evaluations of the submodels included in the AI-based system prototype. **A** Superiority analysis comparing the discrimination performance of each submodel with that of their corresponding ovarian reserve marker models which were developed using a similar approach (strategy deployment models additionally incorporated the four ovarian stimulation components). Age, AFC, AMH, and FSH models incorporated age, AFC, AMH, and FSH only, respectively. D1, D2, and D3 models incorporated age + FSH, age + AFC, and age + AMH, respectively. T1 and T2 models incorporated age + AMH + FSH and age + AMH + AFC, respectively. Q models incorporated age + AMH + FSH + AFC. LORRM and LORSM indicate the risk prediction and strategy submodels for low ovarian response, respectively. HORRM and HORSM indicate the risk prediction and strategy submodels for hyper ovarian response, respectively. **B** Performances of discrimination and calibration of the submodels in the external validation cohort. **C** Sensitivity and subgroup analyses representing various constrained conditions of the external validation cohort

The contribution weights of top important variables included in the AI-based submodels are graphically shown in Fig. 3A. They present a similar spectrum in predicting LOR and HOR except for several unique ones. Shared features also exhibited different contribution profiles. How key risk factors drive the predictions of risk submodels was evaluated as shown in Fig. 3B and C. In general, lower values will drive the predictions toward either LOR/HOR or non-LOR/non-HOR and higher values will drive the predictions in the opposite direction. The correlation analysis among top predictive features revealed that most feature pairs exhibited weak correlations (|*r*|< 0.30), with the strongest correlation observed between AMH and basal AFC (*r* = 0.50), supporting the validity of individual SHAP value interpretations (Additional file 1: Fig. S4).

#### Role of conventional indicators

Conventional ORMs, including age, AMH, AFC, and FSH, combined with the four schedulable COS components, only accounted for an approximate 62.6% the total contribution weight in predicting LOR (ORMs: 41.8%; COS components: 20.8%) and 73.2% in predicting HOR (ORMs: 58.5%; COS components: 14.7%) (Additional file 2: Table S5). As depicted in Fig. 3A, AFC and FSH contributed more to the predictions of HOR compared with LOR. Among four schedulable components, COS protocol showed the largest impact on the LOR risk, followed by recombinant FSH use; while use of recombinant FSH turned to contribute most to the risk of HOR, followed by COS protocol. The significance of FSH starting dose only ranked third in importance for predicting both outcomes. LH supplementation only demonstrated a slight effect on predicting the risk of HOR. For conventional indicators, as expected, advanced age, lower AMH levels and AFC, and higher FSH levels drove risk predictions toward LOR and non-HOR.

#### Beyond conventional indicators

In addition, some unexpected indicators are also relevant to the prediction of LOR and HOR (Fig. 3B and C). An increased diastolic blood pressure, alanine aminotransferase, and red blood cell count drove risk predictions toward LOR, while a decreased white blood cell count drove the prediction toward LOR. Decreased platelet count also drove the prediction toward HOR. Exceptions also exist. For instance, both low and high body weight or basal LH levels were found to influence LOR predictions variably across patients, emphasizing the complexity of ovarian response dynamics.

### Local explanations of the AI-based models

**Fig. 4:**
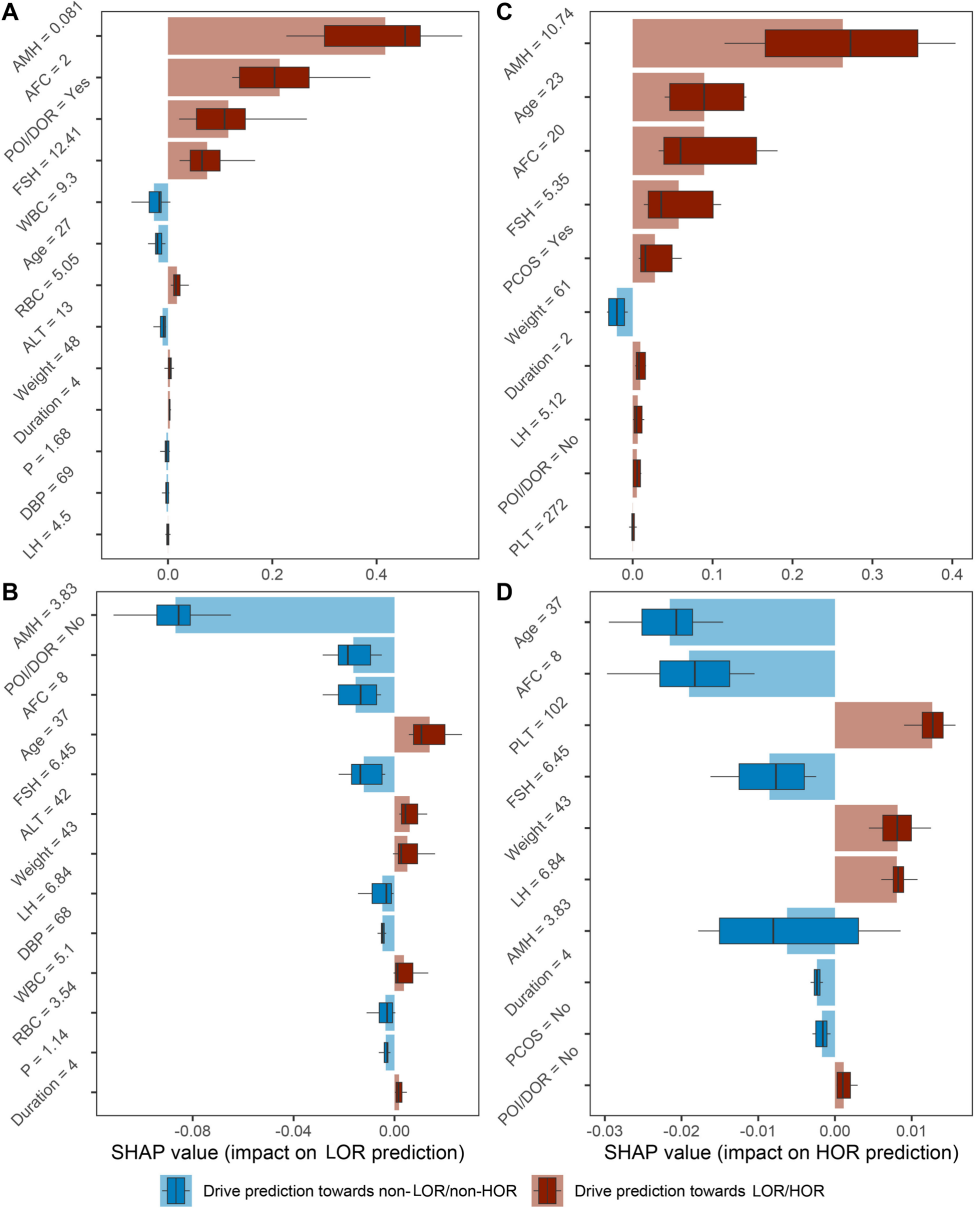
Local interpretability of the risk prediction submodels. How risk factors drove patient-specific predictions were visualized using representative cases. **A** LORRM predicting an actual LOR patient (predicted LOR risk: 93%, predicted incident: LOR); **B** HORRM predicting an actual HOR patient (predicted HOR risk: 62%, predicted incident: HOR); and both the LORRM (**C**) and HORRM (**D**) predictions on the same patient with an actual normal ovarian response (predicted LOR risk: 3%, predicted incident: non-LOR; predicted HOR risk: 6%, predicted incident: non-HOR). Light-colored boxes indicate average SHAP values computed under 100 different feature orderings. Dark-colored boxes and the lines inside indicate the median (IQR). The left and right whiskers indicate the 12.5th and 87.5th percentiles, respectively

At an individual level, how risk factors impacted the patient-specific risk was inspected using four real representative cases (Fig. 4). These effects explain how the features integrated in the AI-based models predicted an individual risk. AMH is the most important feature in three out of four cases (Fig. 4A–C), except for HORRM predicting the individual patient with actual normal ovarian response (Fig. 4D). In this case, a decreased platelet count drives the prediction toward HOR and contributes more than AMH. Generally, key risk factors vary largely in their relative importance and their impact on the direction of risk predictions for different individuals. For the clinical usability of the AI-based system, an example implementation methodology of user-friendly application was described in Additional file 3: Supplementary methods.

### Capability of the AI-based system proposing individualized ovarian stimulation strategies

**Fig. 5:**
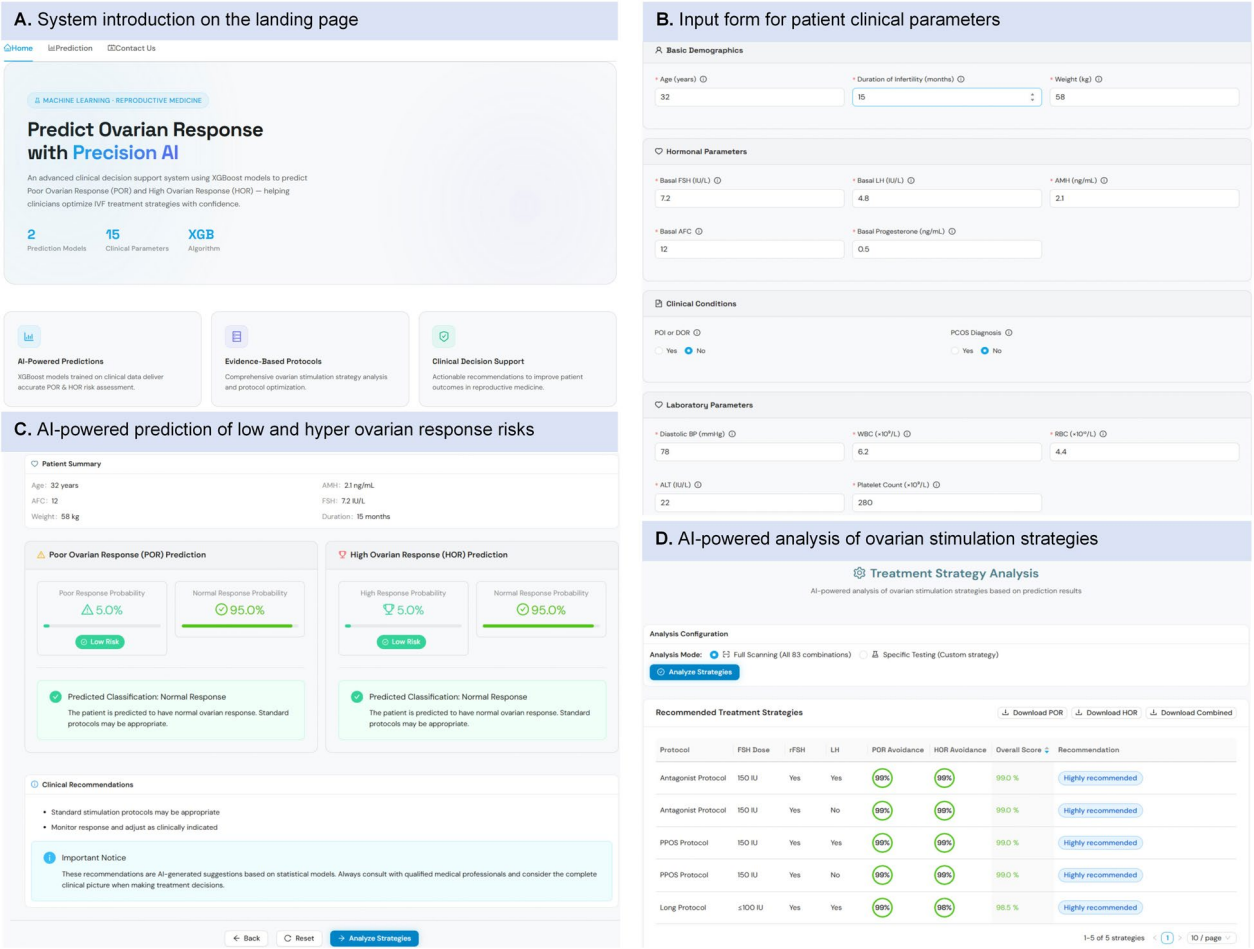
An example of the implementation of the AI-based system prototype. **A** System introduction on the landing page. **B**. Input form for patient clinical parameters. **C** AI-powered prediction output showing estimated risks of low and hyper ovarian response (demonstrated with a sample case). **D** AI-powered analysis of ovarian stimulation strategies based on prediction results (demonstrated with a sample case)

To facilitate clinical application, we developed an interactive web application (https://predict-ovarian-response-web.vercel.app/) that integrates patient data entry, AI-based LOR/HOR risk prediction, and strategy analysis for individualized treatment recommendations (Fig. 5). The capacity of the AI-based strategy submodels to propose effective COS strategies, which encompass combinations of the four schedulable components, was evaluated and summarized in Additional file 2: Table S6. When retrospectively assessed in the independent cohort, both LORSM and HORSM achieved precision exceeding 95% for identifying effective ovarian stimulation strategies (LORSM: 95.5% [94.6%–96.4%]; HORSM: 98.4% [98.0%–98.9%]). Balanced accuracy metrics also reflected high performance, with values of 86.1% (95% CI: 83.4%–88.4%) for LORSM and 85.1% (95% CI: 83.3%–87.1%) for HORSM, alongside Matthews correlation coefficients of 0.80 (95% CI: 0.75–0.84) for LORSM and 0.76 (95% CI: 0.72–0.79) for HORSM. These strategy submodels were also effective in minimizing the inclusion of ineffective COS strategies, with true positive rates of 82.1% (95% CI: 81.0%–83.2%) for LORSM and 87.5% (95% CI: 84.3%–90.7%) for HORSM.

## Discussion

We developed and validated an interpretable and automated AI-based system prototype which showed significant superiority over conventional ovarian reserve indicators in predicting risks of abnormal responses and deploying individualized COS strategies, as well as stable performance in external validation and various constrained conditions. We observed ovarian response-specific risk factors and substantial differences in the relative importance and contribution of baseline factors and COS components to the overall risk among low responders and hyper responders, questioning the assumption that LOR and HOR were the simple transition on the number of oocytes retrieved. In a retrospective evaluation, the strategy submodels demonstrated potential effectiveness in deploying COS strategies. These findings substantiate the advantages of AI in improving the precision of early detection of abnormal ovarian responses and deployment of individualized COS treatment strategies.

IVF is expensive and invasive, as such, early identification of abnormal ovarian response and individualization of COS treatments are of great significance to minimize risks of IVF failure and complications [44, 45]. Recently, Xu et al. built an online strategy model to direct individualized FSH doses for GnRH antagonist OS cycles [46, 47]. Other previous studies have also used classical statistical techniques to establish prediction tools [10–14, 48–51], but none has achieved desired level of generalizability or effectiveness. We assume the current difficulties mainly derive from (1) the variability of individual characteristics, (2) unclear optimal combinations and weights of ORMs for ovarian response assessment, (3) the complexity of COS strategies, such as various protocols, long treatment period, plenty of types and dosing choices of gonadotropin [45], and (4) being limited to small sample sizes or specific scenarios (e.g., a specific COS protocol or predefined population, only focus on FSH dosing). If taking all these into account, compared to classic statistical techniques, AI might be better equipped to handle the inherent assumptions, nonlinear relationships, and the explosions of variables and interactions. In twenty-first century, uses of AI have been growing in medical area, including the analysis of medical images [52], detection of drug interactions [53], identification of high-risk patients [54], and emerging application of large language models (e.g., ChatGPT 5, BioBERT, and NYUTron) [55–57]. However, its performance relative to current practices, as well as the veracity, robustness, and transparency of the answers, needs to be addressed to realize its full potential [58]. Especially in the era of the AI rapid advancement, these efforts are particularly valuable. Although this study is also unable to overcome these challenges, we used the largest sample size of real-world patient data and most comprehensive evaluations to date to empower the AI models. A most comprehensive variable screen can also avoid the exclusion of potential risk factors from the outset. In our study, the interpretation of contribution profiles of certain variables as assigned may also help enhance clinicians’ ability to make informed decisions regarding patients’ physical status.

In most cases, taking oocyte yield as continuous variable seemed to achieve unsatisfactory performance [59–61]. This could be explained by the different prevalence of LOR and HOR, and different physical status or diseases related to them as well (e.g., POI and DOR for LOR, polycystic ovarian syndrome for HOR). In this study, we observed large differences in the contribution profiles of shared ORMs to the LOR risk versus HOR risk. For example, AMH and AFC contribute much more to HOR predictions compared to LOR predictions. Among the four schedulable components, COS protocol and use of recombinant FSH contributed differently with respect to LOR and HOR. We also observed a much larger contribution of LH supplementation to LOR risk compared with HOR, which was consistent with previous findings [21]. Moreover, there were some unique factors that had impacts on the risks of LOR and HOR, respectively, independent of conventional ORMs. Once these markers are added, the predictive efficiency of the model increases significantly. This implied that LOR and HOR might have deeper and unique pathophysiologic mechanisms and thus should be assessed separately and treated with respective wisdoms of COS strategies, rather than directly considering them as a transition pattern on oocyte yield. Separate binary models for LOR and HOR demonstrated superior predictive performance and better also reflect their distinct underlying mechanisms compared to a multiclass model. In clinical application, simultaneous use of both models ensures patients are considered normal responders only when neither risk is identified. This notion is further supported by the observation that, in the external validation cohort, the LORRM and HORRM produced completely non-overlapping positive predictions (0/5702 patients classified as high risk by both models), suggesting that the two conditions occupy distinct regions of the clinical feature space.

There has been an ongoing debate on gonadotropin dosing, albeit emerging evidence building toward a greater preference for mild stimulation for all ovarian responders [17, 62–65], and no expected differences in efficacy for outcomes between individualized FSH dosing and standard dosing [16, 50, 51, 66, 67]. In other words, administering different doses of gonadotropin may result in minimal variations among all categories of ovarian responders. We found FSH starting dose only ranked third in importance for both LOR and HOR risks, after COS protocol and use of recombinant FSH. Rather than placing excessive emphasis on personalized FSH dosages, it may be time to focus on achieving individualization of the entire COS journey and refining it for a balance of efficacy and safety, utilizing the emerging AI technologies.

Interestingly, we discovered several metrics that had never been reported in previous models in the prediction of ovarian response, and by incorporating these metrics, we were able to greatly improve the predictive efficacy of the model. These newly identified metrics may play a role in ovarian response, indicating that we should consider these characteristics while deciding COS strategies. First, we found higher diastolic blood pressure may be associated with an increased risk of LOR. Till now, the relationship between DBP and ovarian response is not well-established, although some previous studies appear to have provided clues for such an association. Previous studies have indicated the association of the cardiovascular disease development with early menopause and POI [68–70]. Young women with POF were found to show marked impairments in carotid intima–media thickness and diastolic performance, changes thought to reflect underlying deficits in endothelial function and circulating endothelial progenitor cells [71]. Another longitudinal study found mean changes of diastolic blood pressure per year in low AMH group were greater than in high AMH group [72]. Additionally, research has shown that POR patients exhibit elevated ovarian microvascular resistance [73]. Microvascular stiffening driven by endothelial dysfunction may concurrently raise diastolic pressure and blunt ovarian responsiveness, a link that remains to be mechanistically defined. Second, we found higher alanine aminotransferase drove risk predictions toward LOR. Consistent with our findings, it has been reported by Wang et al. that the levels of alanine aminotransferase were significantly higher in patients with POI and positively correlated with FSH [74]. Women with POI and subclinical POI were also at risk for liver function impairment. Thus, elevated levels of alanine aminotransferase could be a sign of a potential LOR, which should be taken into consideration in COS. Third, we found higher red blood cell count and decreased white blood cell count drove risk predictions toward LOR. Although no direct relationship between red blood cell counts and ovarian reserve has been found, neutrophil to lymphocyte ratio was reported to be a marker for the diagnosis of POI [75]. A previous study found white blood cell counts and alanine aminotransferase had a positive correlation in POI patients, indicating a possible risk of elevated levels of white blood cell [74]. However, these variables may exert context-dependent effects detectable only through machine learning’s capacity to model interactions.

We also observed that low platelet counts may be associated with an increased risk of HOR. Although there is no prior evidence for this, the platelet-derived growth factors are proved lower in those with increased risk of ovarian hyperstimulation syndrome [76]. Nevertheless, we emphasize that these potential markers identified in this study were just inspired from the AI approaches and that is far from sufficient to unravel the internal relationships. Further clinical or mechanism studies are still needed.

A critical next step for the practical application of the AI-based system is the integration with the information system, which will not only improve accessibility and real-world application but also allow for easier and automated data capture of the patient attributes and treatment outcomes. With research evidence and more diversified data from other centers complemented continuously, the AI-based submodels will be kept updated and enhanced (Additional file 1: Fig. S5). The use of application programming interface will allow for storing the system on a secure cloud-computing server, implementation in different terminals, and improvement in friendliness of the user interface.

This study has several limitations. We did not make comparisons between the performance of the submodels with that of established models. After a thorough literature search, we did not find a study including a general population and the same COS components as ours, which we considered unfair to make comparisons. Besides, we did not directly predict the oocyte yield but rather classified it into LOR and HOR. In fact, initially, we directly predicted the oocyte yield and calculated the root mean square error (RMSE). Consistent with previous literature, the predictive performance was poor, with an RMSE as high as 3.5 (data not shown). RMSE represents the difference between predicted values and actual observed values, serving as a standard deviation of prediction errors. An RMSE of 3.5 is acceptable for predicting HOR but catastrophic for identifying low responders. For example, when the predicted oocyte yield is 4, its actual range spans from 0.5 to 7.5 standard deviations. Therefore, the direct prediction of oocyte yield proves ineffective for identifying LOR. This poor performance also reflects that LOR and HOR do not merely represent a straightforward transition in terms of oocyte yield. Instead, they may involve distinct underlying mechanisms, suggesting that a “segmentation” concept should be employed in COS treatment to avoid their occurrence, rather than solely considering the transitional nature of oocyte yield. Moreover, this study involved two centers from China and bias due to ethnicities cannot be excluded. At this point, caution should be noted when extrapolating any findings from this study to other ethnicities. Additionally, we have not linked our predictions to cumulative live birth rate. While optimizing oocyte yield is foundational, future prospective studies should evaluate whether AI-guided strategies improve downstream reproductive outcomes. Another limitation of our two-model approach is the theoretical potential for contradictory predictions. In the external validation cohort, no patients (0/5702, 0.00%) were simultaneously classified as high risk by both models, consistent with the opposing effects of shared key features on LOR and HOR risk. Nevertheless, borderline contradictions remain theoretically possible. To address this, the CDSS should implement a conflict detection mechanism that presents both probabilities, highlights the higher-probability classification as the primary risk, and flags the case for clinical review. Our cohorts consisted exclusively of Han Chinese women. We would appreciate the addition of new cohort data from different countries and ethnicities and the replication and validation of our results to strengthen the models with better performance and generalizability. Although we retrospectively assessed the ability of strategy submodels proposing COS strategies, the effectiveness cannot be fully stated until large prospective studies for validation are conducted. Last, the validation of certain COS components—particularly recombinant versus urinary FSH—may be limited by practice pattern differences between institutions. Nevertheless, the maintained AUCs of 0.84–0.88 in external validation suggest the models are not overly reliant on this single component. Additionally, an inherent limitation of our strategy models is the joint modeling of patient characteristics and physician-controlled COS decisions. Since physicians select protocols and doses based on their assessment of ovarian reserve and other factors, our models function as “what-if” simulators rather than causal estimators. Future studies employing causal inference frameworks or prospective randomized designs would be valuable to disentangle the independent effects of COS components from patient-level confounders.

## Conclusions

We developed and validated an AI-based system prototype that accurately identifies abnormal ovarian responders and automatically deploys individualized treatment strategies before the commencement of COS. Our findings revealed that while low responders and hyper responders share a comparable profile of risk factors, these factors have distinctly different impacts on overall risk. This variation challenges the traditional belief that LOR and HOR are simply transitions along a continuum of oocyte yield. Beyond conventional ovarian reserve markers, the response-specific indicators and varying influence COS components underscore the complexity of predicting ovarian responses. This study not only substantiates the potential of AI applications in personalized medicine but also serves as a model for how to construct, evaluate, and implement AI models in clinical settings transparently and effectively. Such comprehensive evaluation is particularly valuable given the impending AI rapid advancement and their increasing adoption in healthcare.

## Supplementary Information

Additional file 1: Supplementary figures. Fig. S1 Flowchart of sample sizes corresponding to different modeling processes and analyses. Fig. S2 Performances of discrimination and calibration of candidate machine learning algorithms. Fig. S3 Feature selection process based on the decline rates of the mean absolute SHAP value as the ranking number of features increased. Fig. S4 Correlation matrix of the top predictive features. Fig. S5 Overview of future deployment of the AI-based decision support system.

Additional file 2: Supplementary tables. Table S1 Candidate covariates initially extracted and included in this study. Table S2 Key tools used in this study. Table S3 Distribution of variables with missingness comparing complete data from the derivation cohort with the combined imputed datasets. Table S4 Optimized hyperparameters of the main modeling processes in this study. Table S5 Mean absolute SHAP values and contribution weights of the 55 features in predicting abnormal ovarian response. Table S6 Evaluations of the capacity of strategy submodels to propose effective ovarian stimulation strategies.

Additional file 3: Supplementary methods. Detailed descriptions of standard ovarian stimulation process and drug administration, diagnostic criteria of reproductive disorders, AI algorithms (XGBoost, SVM with RBF, random forest, and MLP), hyperparameter optimization, feature selection and explainable predictions, superiority and sensitivity analyses, and deployment of individualized COS strategies using the AI-based system.

## Data Availability

The datasets used and analyzed during this study are not publicly available due to patient privacy but are available from the corresponding author on reasonable request. The analysis code is available at https://github.com/Guiquan-27/Predict_OR. A web application implementing the AI-based system is accessible at https://predict-ovarian-response-web.vercel.app/.

## Abbreviations

AFC: Antral follicle count
AI: Artificial intelligence
AMH: Anti-Müllerian hormone
AUC: Area under the curve
COS: Controlled ovarian stimulation
DOR: Diminished ovarian reserve
FSH: Follicle-stimulating hormone
GnRH: Gonadotropin-releasing hormone
HOR: Hyper ovarian response
IVF: In vitro fertilization
LASSO: Least Absolute Shrinkage and Selection Operator
LH: Luteinizing hormone
ORMs: Ovarian reserve markers
POI: Primary ovarian insufficiency
LOR: Low ovarian response
RMSE: Root mean square error
SHAP: Shapley additive explanations
XGBoost: Extreme gradient boosting
