# Supplementary material for: Enhanced predictive performance of artificial intelligence in individualized ovarian stimulation of in vitro fertilization: a retrospective cohort study
Source: BMC Med. 2026 Mar 10;24:250. doi: 10.1186/s12916-026-04769-0 (PMC13085636; doi:10.1186/s12916-026-04769-0)
Supplement: Supplementary file 1 — Additional file 1: Supplementary figures. Fig. S1 Flowchart of sample sizes corresponding to different modeling processes and analyses. Fig. S2 Performances of discrimination and calibration of candidate machine learning algorithms. Fig. S3 Feature selection process based on the decline rates of the mean absolute SHAP value as the ranking number of features increased. Fig. S4 Correlation matrix of the top predictive features. Fig. S5 Overview of future deployment of the AI-based decision support system. [file 12916_2026_4769_MOESM1_ESM.docx]

**Supplementary Fig. S1** Flowchart of sample sizes corresponding to different modeling processes and analyses


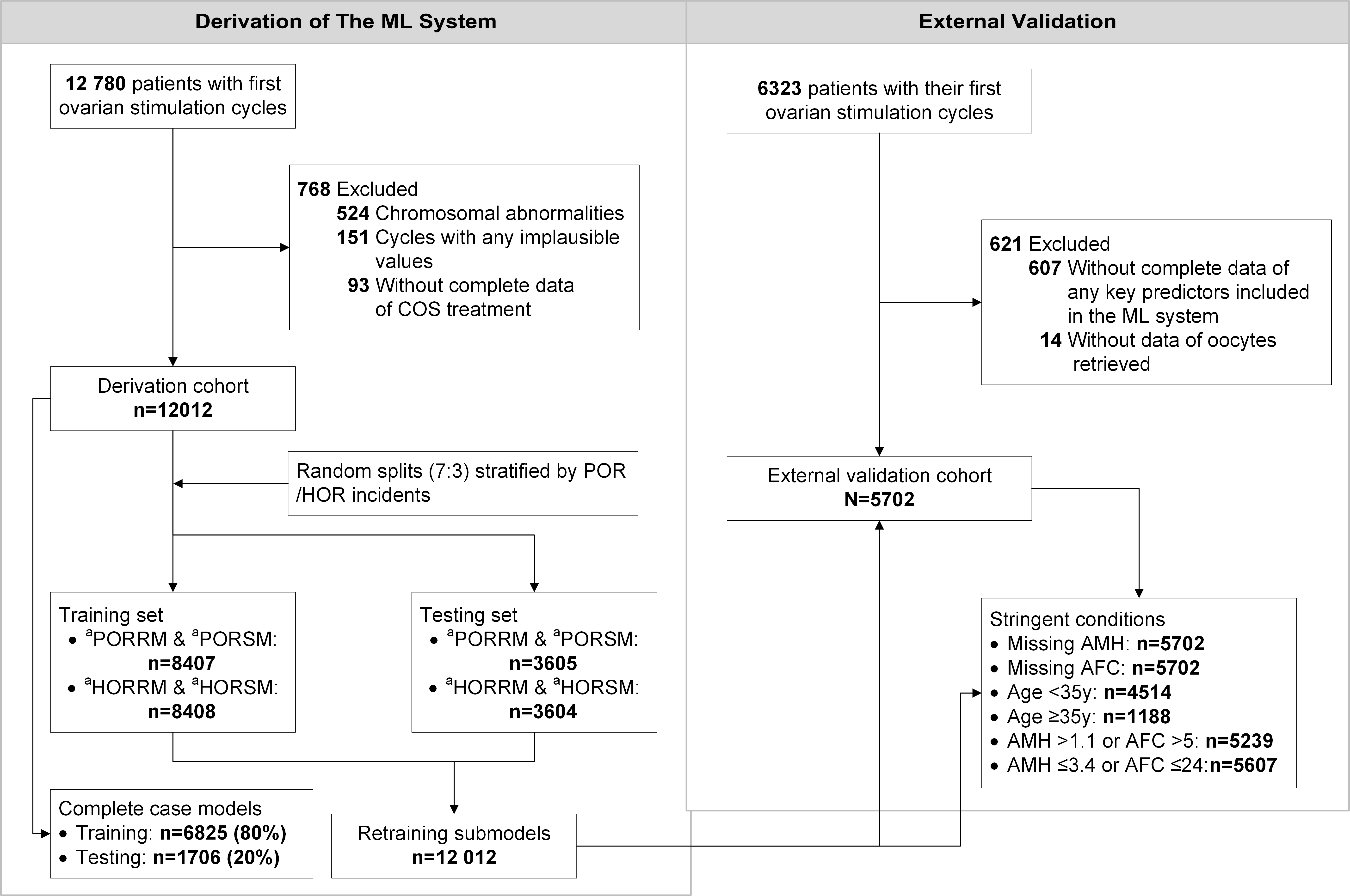


^a^ Candidate risk prediction or strategy models that were developed for different analyses: (1) identification of the best-performing machine learning algorithm, (2) selection of top important features, (3) internal validation and (4) superiority analysis.

Abbreviations: HOR, hyper ovarian response; HORRM, risk prediction models of hyper ovarian response; HORSM, models for strategy deployment of hyper ovarian response; COS, controlled ovarian stimulation; LOR, poor ovarian response; PORRM, risk prediction models of poor ovarian response; PORSM, models for strategy deployment of poor ovarian response.

**Supplementary Fig. S2** Performances of discrimination and calibration of candidate machine learning algorithms


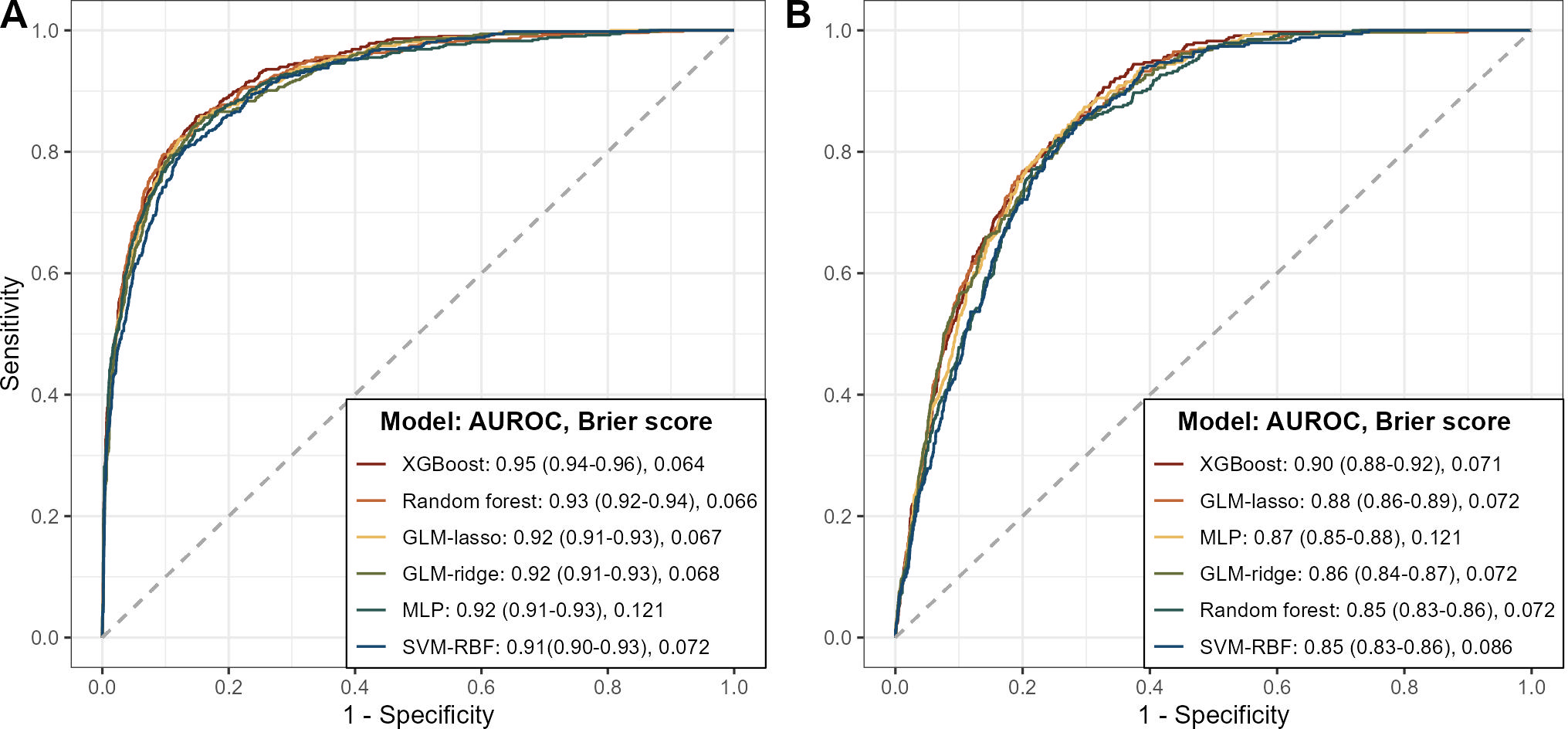


Receiver operating characteristic curves and Brier scores of full-variable strategy deployment models (incorporating all 55 covariates) developed by six representative machine learning algorithms. A. Strategy deployment models of poor ovarian response. B. Strategy deployment models of hyper ovarian response.

**Supplementary Fig. S3** Feature selection process based on the decline rates of the mean absolute SHAP value as the ranking number of features increased


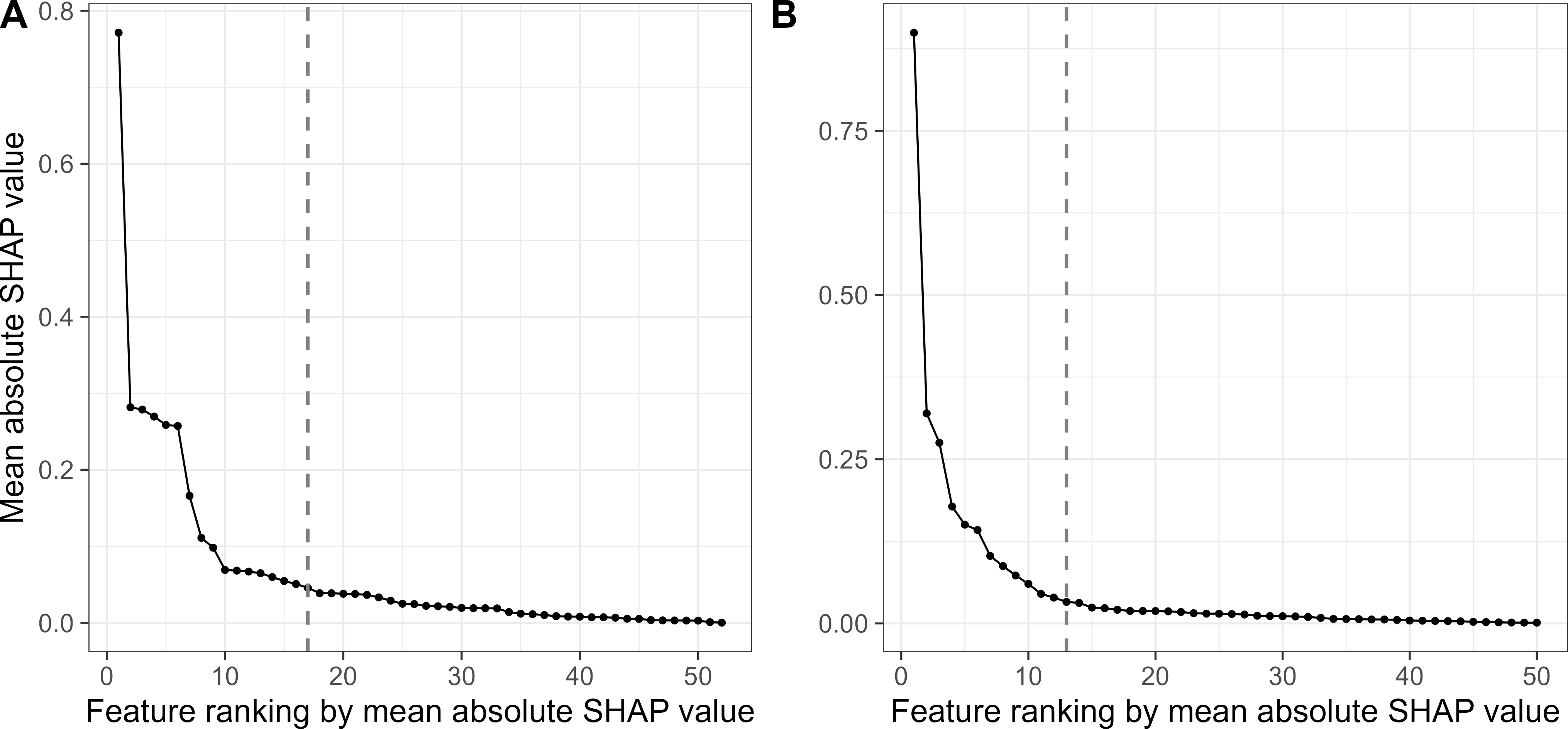


A. Seventeen top important features were selected through calculating and ranking the mean absolute SHAP values of features from the full-variable LOR strategy model incorporating 55 features (3 features were not displayed due to their mean absolute SHAP values equaling to 0). B. Thirteen top features were selected through calculating and ranking the mean absolute SHAP values of features from the full-variable HOR strategy model incorporating 55 features (5 features were not presented). Although the exogenous luteinizing hormone supplementation did not appear in the top predictors for predicting HOR, it was also incorporated to predict HOR due to its importance in ovarian stimulation.

Abbreviations: SHAP, Shapley additive explanations.

**Supplementary Fig. S4** Correlation matrix of the top predictive features

**
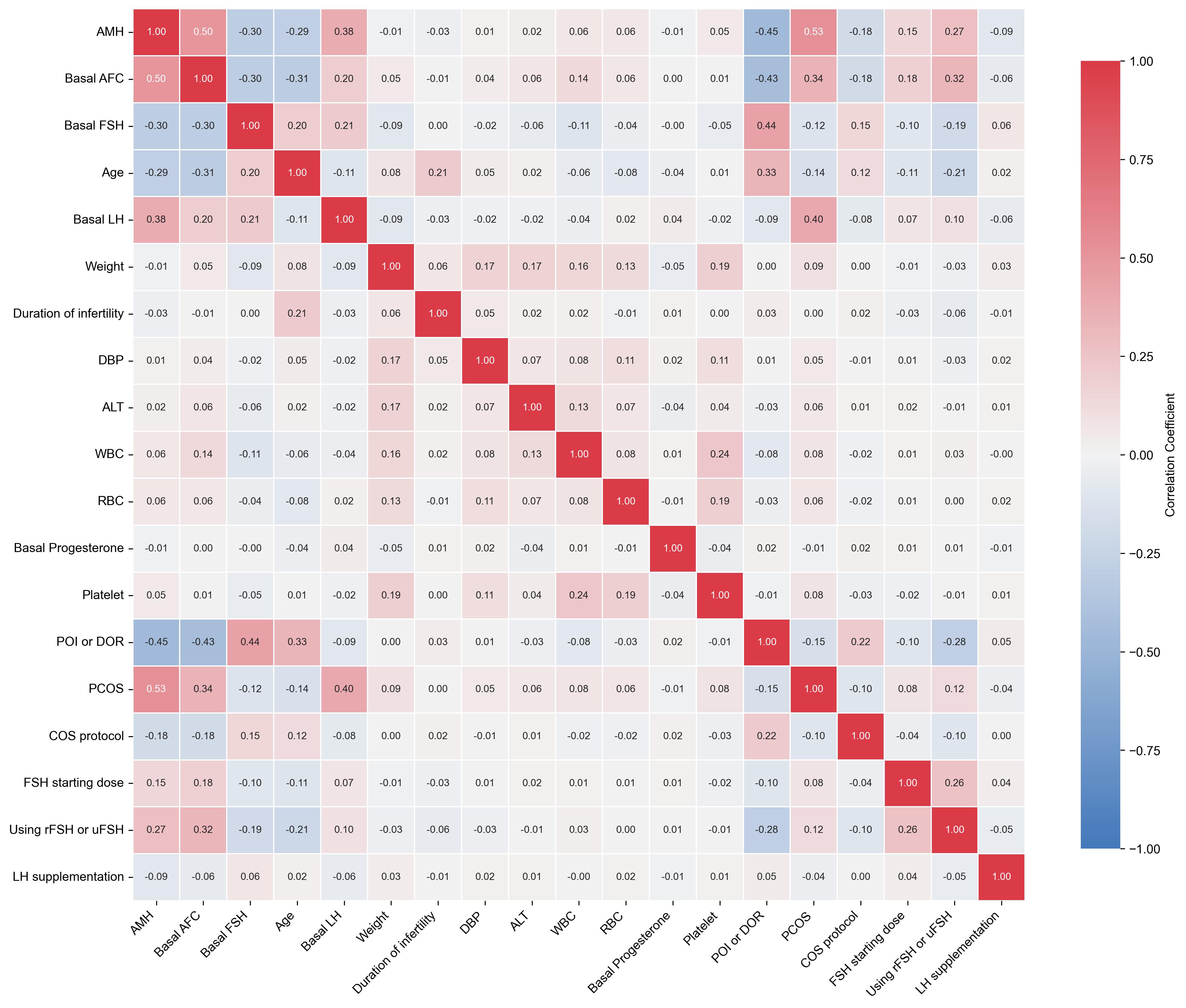
**

Heatmap displaying pairwise Pearson correlation coefficients among the most important predictors identified by SHAP analysis in the LOR and HOR prediction models, along with 4 controlled ovarian stimulation intervention components. Color scale ranges from −1 (blue, negative correlation) to +1 (red, positive correlation), with values within cells indicating correlation coefficients.

**Supplementary Fig. S5** Overview of future deployment of the AI-based decision support system


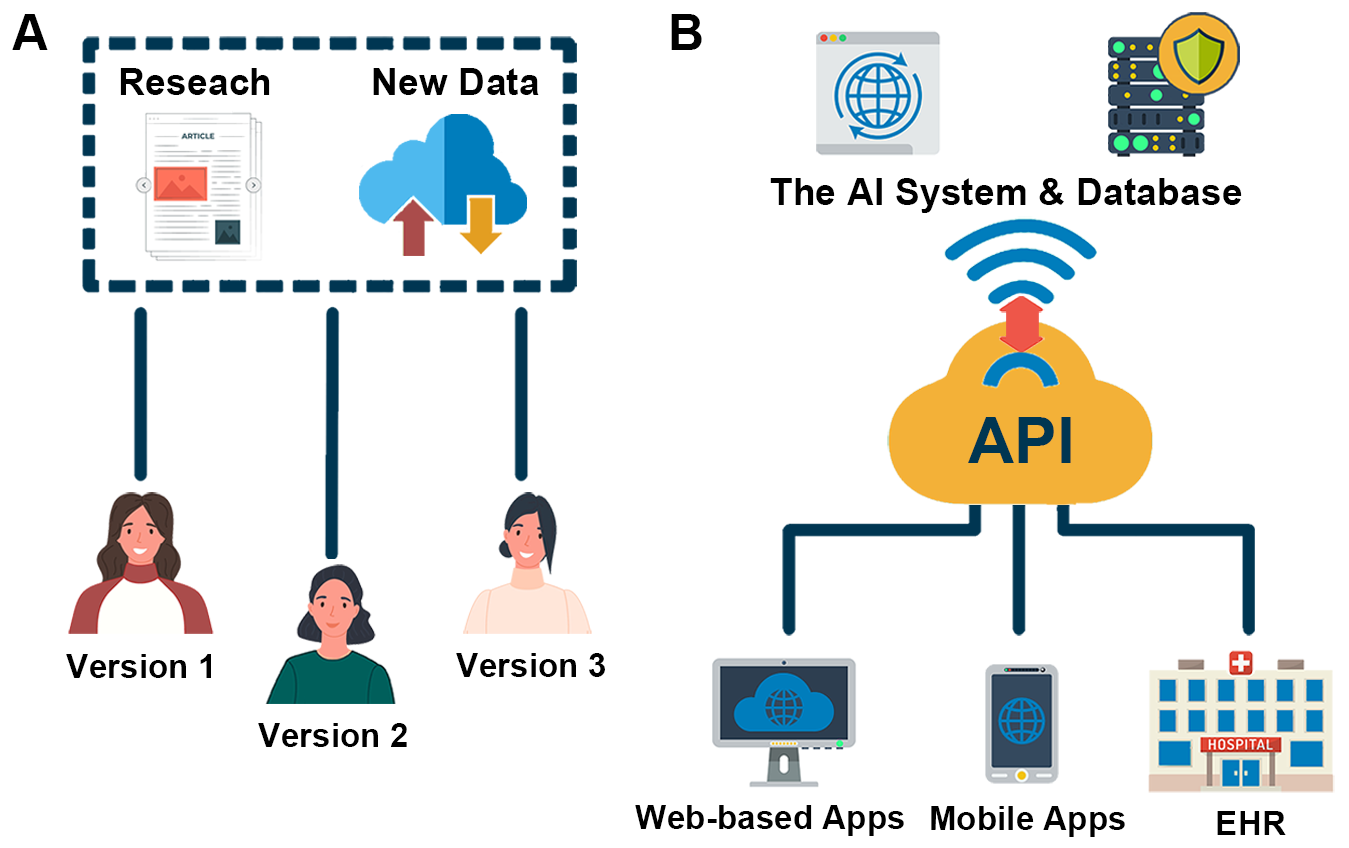


Abbreviations: AI, artificial intelligence; API, application programming interface; EHR, electronic health records.
