# Supplementary material for: Enhanced predictive performance of artificial intelligence in individualized ovarian stimulation of in vitro fertilization: a retrospective cohort study
Source: BMC Med. 2026 Mar 10;24:250. doi: 10.1186/s12916-026-04769-0 (PMC13085636; doi:10.1186/s12916-026-04769-0)
Supplement: Supplementary file 2 — Additional file 2: Supplementary tables. Table S1 Candidate covariates initially extracted and included in this study. Table S2 Key tools used in this study. Table S3 Distribution of variables with missingness comparing complete data from the derivation cohort with the combined imputed datasets. Table S4 Optimized hyperparameters of the main modeling processes in this study. Table S5 Mean absolute SHAP values and contribution weights of the 55 features in predicting abnormal ovarian response. Table S6 Evaluations of the capacity of strategy submodels to propose effective ovarian stimulation strategies. [file 12916_2026_4769_MOESM2_ESM.docx]

**Supplementary Table S1.** **Candidate covariates initially extracted and included in this study**

| **Domain** | **Variable** | **Percentage of Missingness** | **Type** | **Inclusion** |
| --- | --- | --- | --- | --- |
| Demographics | Age | 0 | Numerical | Included |
| Vitals | Diastolic blood pressures | 1 | Numerical | Included |
| Vitals | Systolic blood pressure | 1 | Numerical | Included |
| Vitals | Weight | <1 | Numerical | Included |
| Vitals | Body mass index | <1 | Numerical | Excluded, correlated with weight |
| Ultrasound | Basal antral follicle count | 6 | Numerical | Included |
| Social history | Education level | <1 | Polytomous | Included |
| Social history | Season when oocyte retrieval was finished | 0 | Polytomous | Included |
| Social history | Smoker | 1 | Polytomous | Excluded due to large imbalance |
| Laboratory | Fasting plasma glucose | 52 | Numerical | Excluded due to missingness |
| Laboratory | Low density lipoprotein cholesterol | 51 | Numerical | Excluded due to missingness |
| Laboratory | High density lipoprotein cholesterol | 51 | Numerical | Excluded due to missingness |
| Laboratory | Triglyceride | 51 | Numerical | Excluded due to missingness |
| Laboratory | Total cholesterol | 51 | Numerical | Excluded due to missingness |
| Laboratory | Fasting insulin level | 50 | Numerical | Excluded due to missingness |
| Laboratory | Basal prolactin | 31 | Numerical | Excluded due to missingness |
| Laboratory | Basal androstenedione | 30 | Numerical | Excluded due to missingness |
| Laboratory | Cancer antigen 125 | 24 | Numerical | Excluded due to missingness |
| Laboratory | Homocysteine | 23 | Numerical | Excluded due to missingness |
| Laboratory | Serum urea nitrogen | 20 | Numerical | Excluded due to missingness |
| Laboratory | Anti-Müllerian hormone | 15 | Numerical | Included |
| Laboratory | Basal progesterone | 8 | Numerical | Included |
| Laboratory | Basal estradiol | 7 | Numerical | Included |
| Laboratory | Basal follicle-stimulating hormone | 4 | Numerical | Included |
| Laboratory | Basal luteinizing hormone | 4 | Numerical | Included |
| Laboratory | Red blood cell count | 3 | Numerical | Included |
| Laboratory | White blood cell count | 3 | Numerical | Included |
| Laboratory | PCR detection of *Neisseria gonorrhoeae* | 2 | Dichotomous | Excluded due to large imbalance |
| Laboratory | Hematocrit | 2 | Numerical | Included |
| Laboratory | PCR detection of *Chlamydia trachomatis* | 2 | Dichotomous | Included |
| Laboratory | PCR detection of *Ureaplasma urealyticum* and *Mycoplasma hominis* | 1 | Dichotomous | Excluded due to large imbalance |
| Laboratory | Serum creatinine | 1 | Numerical | Included |
| Laboratory | Prothrombin time | 1 | Numerical | Included |
| Laboratory | Activated partial thromboplastin time | 1 | Numerical | Included |
| Laboratory | ABO blood type | <1 | Polytomous | Included |
| Laboratory | Albumin | <1 | Numerical | Included |
| Laboratory | Aspartate aminotransferase | <1 | Numerical | Included |
| Laboratory | Serum total protein | <1 | Numerical | Included |
| Laboratory | Rhesus Macacus blood type | <1 | Dichotomous | Excluded due to large imbalance |
| Laboratory | Alanine aminotransferase | <1 | Numerical | Included |
| Laboratory | Platelet count | <1 | Numerical | Included |
| Laboratory | Hemoglobin | <1 | Numerical | Included |
| Clinical history | Duration of infertility | 5 | Numerical | Included |
| Clinical history | Frequency of menstrual cycles | 1 | Polytomous | Included |
| Clinical history | Age at menarche | 1 | Numerical | Included |
| Clinical history | Abnormal reproductive history | <1 | Polytomous | Included |
| Clinical history | Regularity of menstrual cycles | <1 | Polytomous | Included |
| Clinical history | Primary/Secondary infertility | <1 | Dichotomous | Included |
| Clinical history | Allergy history | <1 | Dichotomous | Included |
| Clinical history | Penicillin allergic | <1 | Dichotomous | Excluded due to large imbalance |
| Clinical history | Cephalosporin allergic | <1 | Dichotomous | Excluded due to large imbalance |
| Clinical history | Maternal history | <1 | Polytomous | Included |
| Clinical history | Paternal history | <1 | Polytomous | Included |
| Clinical history | Menstrual bleeding volume | <1 | Polytomous | Included |
| Clinical history | Times of abortion | <1 | Polytomous | Included |
| Clinical history | Times of gravidity | <1 | Polytomous | Included |
| Clinical history | Dysmenorrhea | 0 | Dichotomous | Included |
| Clinical history | Parity | 0 | Dichotomous | Included |
| Clinical history | Risk factors for poor ovarian response | 0 | Dichotomous | Included |
| Clinical history | Polycystic ovarian syndrome | 0 | Dichotomous | Included |
| Clinical history | Tubal factor for infertility | 0 | Dichotomous | Included |
| Clinical history | Endometriosis | 0 | Dichotomous | Included |
| Clinical history | Unexplained factor for infertility | 0 | Dichotomous | Included |
| Clinical history | Pelvic inflammatory disease | 0 | Dichotomous | Included |
| Clinical history | Premature ovarian insufficiency or diminished ovarian reserve | 0 | Dichotomous | Included |
| Clinical history | Hypothyroidism | 0 | Dichotomous | Included |
| Clinical history | Male factor for infertility | 0 | Dichotomous | Included |
| Clinical history | Genetic factor | 0 | Dichotomous | Included |
| Clinical history | Recurrent miscarriage | 0 | Dichotomous | Included |
| Clinical history | Hypothyroidism | 0 | Dichotomous | Included |
| Clinical history | Male factor for infertility | 0 | Dichotomous | Included |
| Clinical history | Genetic factor | 0 | Dichotomous | Included |
| Clinical history | Recurrent miscarriage | 0 | Dichotomous | Included |
| COS component | Follicle-stimulating hormone starting dose | <1 | Polytomous | Included |
| COS component | Using recombinant or urinary follicle-stimulating hormone | <1 | Polytomous | Included |
| COS component | Exogenous luteinizing hormone supplementation | <1 | Polytomous | Included |
| COS component | Ovarian stimulation protocol | 0 | Polytomous | Included |
| Outcome | Number of oocytes retrieved | 0 | Numerical | Included |

Abbreviations: COS, controlled ovarian stimulation; PCR, polymerase chain reaction.

**Supplementary Table S2.** **Key tools used in this study**

| **Study procedure** | **Key tools** | **Source** |
| --- | --- | --- |
| Data manipulation | tidyverse^a^ | https://www.tidyverse.org |
| Sample splitting | rsample^a^ | https://rsample.tidymodels.org |
| Multiple imputation | miceRanger^a^ | https://github.com/FarrellDay/miceRanger |
| Hyperparameter tuning, Model development | tidymodels^a^ | https://www.tidymodels.org |
| AUC, DeLong test | pROC^a^ | https://web.expasy.org/pROC/ |
| Feature importance | SHAPforxgboost^a^ | https://liuyanguu.github.io/SHAPforxgboost |
| Local explainability of the ML system | DALEX, DALEXtra^a^ | https://dalex.drwhy.ai  https://modeloriented.github.io/DALEXtra |
| Web-based application implementation | shiny^a^ | http://www.rstudio.com/shiny |
| Data visualization (except for the alluvial plot) | ggplot2, ggpubr, ggforce^a^ | https://ggplot2.tidyverse.org  https://rpkgs.datanovia.com/ggpubr  https://ggforce.data-imaginist.com |
| Alluvial plot | Origin 2022b^b^ | https://www.originlab.com |

^a^ R packages.

^b^ Software.

Abbreviations: AUC, area under the receiver operating characteristic curve.

**Supplementary Table S3. Distribution of variables with missingness comparing complete data from the derivation cohort with the combined imputed datasets (training plus testing) for predicting LOR and for predicting HOR**

| **Feature** | **Missingness, No. (%)** | **Complete case data** | **Combined imputed data for predicting LOR** | **Combined imputed data for predicting HOR** |
| --- | --- | --- | --- | --- |
| AMH, ng/mL | 1830 (15.2) | 2.8 (1.5-4.7) | 2.9 (1.5-4.8) | 2.9 (1.5-4.8) |
| Basal progesterone, ng/mL | 1010 (8.4) | 0.6 (0.4-0.8) | 0.6 (0.4-0.8) | 0.6 (0.4-0.8) |
| Basal estradiol, pg/mL | 852 (7.1) | 35.6 (32.4-38.8) | 35.5 (32.3-38.7) | 35.6 (32.5-38.7) |
| Basal AFC, n | 722 (6.0) | 10 (6-15) | 10 (6-15) | 10 (6-15) |
| Duration of infertility, y | 637 (5.3) | 3 (2-4) | 3 (2-4) | 3 (2-4) |
| Basal FSH, IU/L | 539 (4.5) | 7.3 (6.1–8.9) | 7.3 (6.1–8.9) | 7.3 (6.1–8.9) |
| Basal LH, IU/L | 533 (4.4) | 4.9 (3.6-6.6) | 4.9 (3.6-6.6) | 4.9 (3.6-6.6) |
| Red blood cell count, 10^12^/L | 329 (2.7) | 4.4 (4.2-4.6) | 4.4 (4.2-4.6) | 4.4 (4.2-4.6) |
| White blood cell count, 10^9^/L | 324 (2.7) | 6.6 (5.4-8.0) | 6.6 (5.4-8.0) | 6.6 (5.4-8.0) |
| Hematocrit, proportion of 1.0 | 287 (2.4) | 0.39 (0.37-0.41) | 0.39 (0.37-0.41) | 0.39 (0.37-0.41) |
| PCR detection of *Chlamydia trachomatis*, No. (%) | 200 (1.7) |  |  |  |
| Yes |  | 196 (1.7%) | 201 (1.7%) | 198 (1.6%) |
| No |  | 11616 (98.3%) | 11811 (98.3%) | 11814 (98.4%) |
| Frequency of menstrual cycles, No. (%) | 169 (1.4) |  |  |  |
| Normal |  | 9237 (78.0%) | 9326 (77.6%) | 9318 (77.6%) |
| Less |  | 2554 (21.6%) | 2632 (21.9%) | 2637 (22.0%) |
| More |  | 52 (0.4%) | 54 (0.4%) | 57 (0.5%) |
| Diastolic blood pressure, mmHg | 151 (1.3) | 71 (66-78) | 71 (66-78) | 71 (66-78) |
| Systolic blood pressure, mmHg | 146 (1.2) | 115 (109-120) | 115 (109-120) | 115 (109-120) |
| Serum creatinine, μmol/L | 145 (1.2) | 59.5 (53.2-65.9) | 59.5 (53.2-65.9) | 59.5 (53.2-65.9) |
| Prothrombin time, s | 140 (1.2) | 13.0 (12.6-13.4) | 13.0 (12.6-13.4) | 13.0 (12.6-13.4) |
| Activated partial thromboplastin time, s | 137 (1.1) | 35.1 (33.0-37.3) | 35.1 (33.0-37.4) | 35.1 (33.0-37.3) |
| Age at menarche, y | 136 (1.1) | 14 (13-14) | 14 (13-14) | 14 (13-14) |
| ABO blood type, No. (%) | 116 (1.0) |  |  |  |
| A |  | 3689 (31.0%) | 3728 (31.0%) | 3724 (31.0%) |
| B |  | 3133 (26.3%) | 3163 (26.3%) | 3166 (26.4%) |
| O |  | 4017 (33.8%) | 4056 (33.8%) | 4054 (33.7%) |
| AB |  | 1057 (8.9%) | 1065 (8.9%) | 1068 (8.9%) |
| Albumin | 113 (0.9) | 43.7 (41.9-45.5) | 43.7 (41.9-45.5) | 43.7 (41.9-45.5) |
| Aspartate aminotransferase, U/L | 95 (0.8) | 18.0 (16.0-21.0) | 18.0 (16.0-21.0) | 18.0 (16.0-21.0) |
| Serum total protein, g/L | 93 (0.8) | 72.1 (68.9-75.1) | 72.1 (68.9-75.1) | 72.1 (68.9-75.1) |
| Abnormal reproductive history, No. (%) | 92 (0.8) |  |  |  |
| Yes |  | 3806 (31.9%) | 3812 (31.7%) | 3811 (31.7%) |
| No |  | 8114 (68.1%) | 8200 (68.3%) | 8201 (68.3%) |
| Regularity of menstrual cycles, No. (%) | 79 (0.7) |  |  |  |
| Regular |  | 9720 (81.5%) | 9783 (81.4%) | 9787 (81.5%) |
| Irregular |  | 2213 (18.5%) | 2229 (18.6%) | 2225 (18.5%) |
| Alanine aminotransferase, U/L | 69 (0.6) | 15 (11-21) | 15 (11-21) | 15 (11-21) |
| Platelet, 10^9^/L | 61 (0.5) | 233 (198-273) | 233 (198-273) | 233 (198-273) |
| Hemoglobin, g/L | 59 (0.5) | 130 (124-136) | 130 (124-136) | 130 (124-136) |
| Education level, No. (%) | 17 (0.1) |  |  |  |
| Primary or below |  | 465 (3.9%) | 466 (3.9%) | 465 (3.9%) |
| Junior |  | 4094 (34.1%) | 4102 (34.1%) | 4098 (34.1%) |
| High |  | 3446 (28.7%) | 3449 (28.7%) | 3451 (28.7%) |
| Bachelor |  | 3392 (28.3%) | 3396 (28.3%) | 3400 (28.3%) |
| Master or above |  | 598 (5.0%) | 599 (5.0%) | 598 (5.0%) |
| Primary/Secondary infertility, No. (%) | 11 (0.1) |  |  |  |
| Primary |  | 5894 (49.1%) | 5905 (49.2%) | 5905 (49.2%) |
| Secondary |  | 6107 (50.9%) | 6107 (50.8%) | 6107 (50.8%) |

Numerical variables are reported as median (interquartile range, IQR). Categorical variables are reported as numbers with the percentages in parentheses.

**Supplementary Table S4. Optimized hyperparameters of the main modeling processes in this study**

| **Models** | **Trees** | **Tree depth** | **Learning rate** | **Mtry** | **Min_n** | **Loss reduction** | **Sample size** | **Penalty** | **Cost** | **Rbf_sigma** | **Margin** | **Hidden units** |
| --- | --- | --- | --- | --- | --- | --- | --- | --- | --- | --- | --- | --- |
| Full LOR strategy models |  |  |  |  |  |  |  |  |  |  |  |  |
| GLM-lasso | — | — | — | — | — | — | — | 1.70×10^-3^ | — | — | — | — |
| GLM-ridge | — | — | — | — | — | — | — | 6.97×10^-4^ | — | — | — | — |
| SVM-RBF | — | — | — | — | — | — | — | — | 0.657 | 3.77×10^-3^ | 0.0579 | — |
| Random forest | 923 | — | — | 8 | 36 | — | — | — | — | — | — | — |
| MLP | — | — | — | — | — | — | — | 0.898 | — | — | — | 1 |
| XGBoost | 936 | 7 | 0.00816 | 13 | 20 | 1.12×10^-9^ | 0.71 | — | — | — | — | — |
| Full HOR strategy models |  |  |  |  |  |  |  |  |  |  |  |  |
| GLM-lasso | — | — | — | — | — | — | — | 3.66×10^-3^ | — | — | — | — |
| GLM-ridge | — | — | — | — | — | — | — | 0.157 | — | — | — | — |
| SVM-RBF | — | — | — | — | — | — | — | — | 0.099 | 2.41×10^-3^ | 0.0332 | — |
| Random forest | 979 | — | — | 5 | 27 | — | — | — | — | — | — | — |
| MLP | — | — | — | — | — | — | — | 0.8 |  |  |  | 1 |
| XGBoost | 197 | 6 | 0.0802 | 23 | 37 | 5.8 | 0.83 | — | — | — | — | — |
| PORRMs |  |  |  |  |  |  |  |  |  |  |  |  |
| Using training dataset | 711 | 9 | 0.0113 | 4 | 33 | 8.05×10^-8^ | 0.97 | — | — | — | — | — |
| Using all derivation data | 895 | 11 | 0.00593 | 4 | 19 | 7.30×10^-9^ | 0.6 | — | — | — | — | — |
| Using complete data | 711 | 9 | 0.0113 | 4 | 33 | 8.05×10^-8^ | 0.97 | — | — | — | — | — |
| PORSMs |  |  |  |  |  |  |  |  |  |  |  |  |
| Using training dataset | 784 | 10 | 0.0108 | 5 | 6 | 7.76×10^-6^ | 0.31 | — | — | — | — | — |
| Using all derivation data | 178 | 2 | 0.0829 | 7 | 19 | 1.35×10^-10^ | 0.66 | — | — | — | — | — |
| Using complete data | 784 | 10 | 0.0108 | 5 | 6 | 7.76×10^-6^ | 0.31 | — | — | — | — | — |
| HORRMs |  |  |  |  |  |  |  |  |  |  |  |  |
| Using training dataset | 837 | 2 | 0.0131 | 2 | 28 | 6.23×10^-10^ | 0.64 | — | — | — | — | — |
| Using all derivation data | 588 | 2 | 0.0134 | 4 | 5 | 2.35×10^-10^ | 0.15 | — | — | — | — | — |
| Using complete data | 837 | 2 | 0.0131 | 2 | 28 | 6.23×10^-10^ | 0.64 | — | — | — | — | — |
| HORSMs |  |  |  |  |  |  |  |  |  |  |  |  |
| Using training dataset | 867 | 9 | 0.00745 | 4 | 7 | 0.212 | 0.4 | — | — | — | — | — |
| Using all derivation data | 828 | 12 | 0.00728 | 15 | 6 | 5.06×10^-10^ | 0.11 | — | — | — | — | — |
| Using complete data | 867 | 9 | 0.00745 | 4 | 7 | 0.212 | 0.4 | — | — | — | — | — |

Abbreviations: HORRM and HORSM indicate risk prediction and strategy deployment submodels for predicting hyper ovarian response (HOR), respectively. PORRM and PORSM are those for poor ovarian response (LOR), respectively.

**Supplementary Table S5. Mean absolute SHAP values and contribution weights of the 55 features in predicting abnormal ovarian response**

| **Ranking Number** | **Predicting LOR** | | | **Predicting HOR** | | |
| --- | --- | --- | --- | --- | --- | --- |
|  | **Variable** | **SHAP value^a^** | **Weight (%)** | **Variable** | **SHAP value^a^** | **Weight (%)** |
| 1 | AMH | 0.771320 | 21.82 | AMH | 0.899371 | 32.13 |
| 2 | Basal AFC | 0.281740 | 7.97 | Basal AFC | 0.319809 | 11.43 |
| 3 | POI or DOR | 0.278760 | 7.88 | Basal FSH | 0.275044 | 9.83 |
| 4 | COS protocol | 0.269561 | 7.62 | Using rFSH or uFSH | 0.177931 | 6.36 |
| 5 | Basal FSH | 0.258634 | 7.32 | COS Protocol | 0.150394 | 5.37 |
| 6 | Using rFSH or uFSH | 0.257239 | 7.28 | Age | 0.142213 | 5.08 |
| 7 | Age | 0.165962 | 4.69 | Basal LH | 0.102745 | 3.67 |
| 8 | FSH starting dose | 0.111026 | 3.14 | POI or DOR | 0.087283 | 3.12 |
| 9 | LH supplementation | 0.098152 | 2.78 | FSH starting dose | 0.072994 | 2.61 |
| 10 | Basal progesterone | 0.069111 | 1.95 | PCOS | 0.060231 | 2.15 |
| 11 | Weight | 0.068235 | 1.93 | Platelet count | 0.044962 | 1.61 |
| 12 | Diastolic blood pressure | 0.066974 | 1.89 | Weight | 0.039344 | 1.41 |
| 13 | White blood cell count | 0.064847 | 1.83 | Duration of infertility | 0.032752 | 1.17 |
| 14 | Alanine aminotransferase | 0.059800 | 1.69 | Serum total protein | 0.031234 | 1.12 |
| 15 | Red blood cell count | 0.054672 | 1.55 | Albumin | 0.024132 | 0.86 |
| 16 | Duration of infertility | 0.050731 | 1.43 | Hemoglobin | 0.023259 | 0.83 |
| 17 | Basal LH | 0.045710 | 1.29 | Diastolic blood pressure | 0.020761 | 0.74 |
| 18 | Albumin | 0.038716 | 1.10 | APTT | 0.018980 | 0.68 |
| 19 | Platelet count | 0.038689 | 1.09 | Progesterone | 0.018973 | 0.68 |
| 20 | Systolic blood pressure | 0.037953 | 1.07 | Systolic blood pressure | 0.018857 | 0.67 |
| 21 | Basal estradiol | 0.037700 | 1.07 | Season of treatment time | 0.018352 | 0.66 |
| 22 | Regularity of menstrual cycles | 0.036536 | 1.03 | Basal estradiol | 0.017358 | 0.62 |
| 23 | Prothrombin time | 0.033237 | 0.94 | Regularity of menstrual cycles | 0.015602 | 0.56 |
| 24 | Hematocrit | 0.028953 | 0.82 | Risk factors for LOR | 0.014869 | 0.53 |
| 25 | APTT | 0.024907 | 0.70 | Alanine aminotransferase | 0.014811 | 0.53 |
| 26 | Paternal history | 0.024503 | 0.69 | Serum creatinine | 0.014343 | 0.51 |
| 27 | Serum creatinine | 0.022131 | 0.63 | Frequency of menstrual cycles | 0.013536 | 0.48 |
| 28 | Times of gravidity | 0.021589 | 0.61 | White blood cell count | 0.011748 | 0.42 |
| 29 | Serum total protein | 0.020878 | 0.59 | Male factor for infertility | 0.011203 | 0.4 |
| 30 | Hemoglobin | 0.019464 | 0.55 | LH supplementation | 0.010962 | 0.39 |
| 31 | Abnormal reproductive history | 0.019189 | 0.54 | Aspartate aminotransferase | 0.010630 | 0.38 |
| 32 | Aspartate aminotransferase | 0.019076 | 0.54 | Education | 0.009799 | 0.35 |
| 33 | Season of treatment time | 0.018804 | 0.53 | Red blood cell count | 0.008400 | 0.3 |
| 34 | ABO blood type | 0.014038 | 0.40 | Abnormal reproductive history | 0.006869 | 0.25 |
| 35 | Frequency of menstrual cycles | 0.011965 | 0.34 | Hematocrit | 0.006658 | 0.24 |
| 36 | Allergy history | 0.011316 | 0.32 | Prothrombin time | 0.006457 | 0.23 |
| 37 | Maternal history | 0.010180 | 0.29 | ABO blood type | 0.006048 | 0.22 |
| 38 | Risk factors for LOR | 0.008704 | 0.25 | Age at menarche | 0.005995 | 0.21 |
| 39 | Male factor for infertility | 0.008145 | 0.23 | Tubal factor for infertility | 0.005345 | 0.19 |
| 40 | Pelvic inflammatory disease | 0.008020 | 0.23 | Paternal history | 0.004374 | 0.16 |
| 41 | Age at menarche | 0.007257 | 0.21 | Maternal history | 0.004192 | 0.15 |
| 42 | Primary/Secondary infertility | 0.007244 | 0.20 | Dysmenorrhea | 0.003792 | 0.14 |
| 43 | Education | 0.006667 | 0.19 | Times of gravidity | 0.003493 | 0.12 |
| 44 | Times of abortion | 0.005383 | 0.15 | Parity | 0.003236 | 0.12 |
| 45 | Endometriosis | 0.005164 | 0.15 | Times of abortion | 0.002423 | 0.09 |
| 46 | Unexplained factor for infertility | 0.003564 | 0.1 | Primary/Secondary infertility | 0.001975 | 0.07 |
| 47 | Dysmenorrhea | 0.003172 | 0.09 | Genetic factor for infertility | 0.001608 | 0.06 |
| 48 | Tubal factor for infertility | 0.003089 | 0.09 | Endometriosis | 0.001306 | 0.05 |
| 49 | Menstrual bleeding volume | 0.002974 | 0.08 | Pelvic inflammatory disease | 0.001189 | 0.04 |
| 50 | Parity | 0.002901 | 0.08 | Allergy history | 0.001159 | 0.04 |
| 51 | PCOS | 0.000845 | 0.02 | Recurrent miscarriage | 0 | 0 |
| 52 | Genetic factor for infertility | 0.000211 | 0 | Hypothyroidism | 0 | 0 |
| 53 | Recurrent miscarriage | 0 | 0 | Unexplained factor for infertility | 0 | 0 |
| 54 | Hypothyroidism | 0 | 0 | PCR detection of *Chlamydia trachomatis* | 0 | 0 |
| 55 | PCR detection of *Chlamydia trachomatis* | 0 | 0 | Menstrual bleeding volume | 0 | 0 |

Shaded features were included in the submodels of AI-based system. A 1% difference in contribution weight was considered similar.

^a^ Mean absolute SHAP value.

Abbreviations: APTT, activated partial thromboplastin time.

**Supplementary Table S6. Evaluations of the capacity of strategy submodels to propose effective ovarian stimulation strategies**

| Metrics | Deploy ovarian stimulation strategies for LOR, mean (95% CI) | Deploy ovarian stimulation strategies for HOR, mean (95% CI) |
| --- | --- | --- |
| Internal validation |  |  |
| Precision^a^, % | 97.0 (96.4–97.7) | 98.2 (97.7–98.7) |
| True positive rate, % | 84.3 (81.2–87.3) | 87.4 (83.8–90.8) |
| Balanced accuracy, % | 89.2 (87.5–91.4) | 85.9 (83.9–87.8) |
| MCC | 0.84 (0.80–0.89) | 0.77 (0.74–0.80) |
| External validation |  |  |
| Precision^a^, % | 95.5 (94.6–95.4) | 98.4 (98.0–98.9) |
| True positive rate, % | 82.1 (81.0–83.2) | 87.5 (84.3–90.7) |
| Balanced accuracy, % | 86.2 (83.4–89.1) | 85.1 (83.3–87.1) |
| MCC | 0.80 (0.75–0.84) | 0.76 (0.72–0.79) |

^a^ Precision for negative class (non-LOR/non-HOR), namely, the probability of the ovarian stimulation strategies of non-LOR/non-HOR patients covered in the strategy list that was proposed by the strategy submodels.

Abbreviations: HOR, hyper ovarian response; MCC, Matthews correlation coefficient; LOR, poor ovarian response.
