# Supplementary material for: Enhanced predictive performance of artificial intelligence in individualized ovarian stimulation of in vitro fertilization: a retrospective cohort study
Source: BMC Med. 2026 Mar 10;24:250. doi: 10.1186/s12916-026-04769-0 (PMC13085636; doi:10.1186/s12916-026-04769-0)
Supplement: Supplementary file 3 — Additional file 3: Supplementary methods. Detailed descriptions of standard ovarian stimulation process and drug administration, diagnostic criteria of reproductive disorders, AI algorithms (XGBoost, SVM with RBF, random forest, and MLP), hyperparameter optimization, feature selection and explainable predictions, superiority and sensitivity analyses, and deployment of individualized COS strategies using the AI-based system. [file 12916_2026_4769_MOESM3_ESM.docx]

# Supplementary Methods

## Standard ovarian stimulation process, drug administration and measurements

As for treatment domains, controlled ovarian stimulation (COS) strategies were deployed by each patient’s primary physician (senior attending physician and above with more than 10-year experience in reproductive medicine) based on age, body mass index, and common ovarian reserve markers (such as anti-Müllerian hormone [AMH], basal antral follicle count [AFC] and follicle-stimulating hormone [FSH]). GnRH agonist (including long, ultra-long and short protocols), GnRH antagonist, mild or (modified) natural cycle, and progestin-primed COS protocols were used. The GnRH agonist was used to induce pituitary downregulation through the suppression of gonadotropin release at the beginning of COS treatment. GnRH agonist preparations used in this study included triptorelin (Decapeptyl, Ferring Pharma; Diphereline, Ipsen Pharma Biotech), leuprorelin (Enantone, Takeda Pharma; Beiyi, Livzon Pharma) and goserelin (Zoladex, AstraZeneca Pharma). The GnRH antagonist preparations included cetrorelix (Cetrotide, Baxter Oncology) and ganirelix (Orgalutran, Merck Sharp & Dohme). Exogenous FSH preparations, including recombinant FSH (rFSH) (Gonal-f, Merck Serono; Puregon, Merck Sharp & Dohme; Jintropin, GenSci Phama) and urinary FSH (uFSH) (Lishenbao, Livzon Pharma; Fostimon, IBSA), were administered for the induction and maintenance of growth of multiple dominant follicles. In most cases of this study, the exogenous FSH starting doses varied between 100 and 300 IU/day, as per physician discretion. Different starting doses were divided into 6 categories as follows: ≤ 100, 150, 200, 225, and ≥ 300 IU/day (93% were 300 IU/day) and non-GnRH (neither GnRH agonist nor antagonist protocols were used). The supplemented exogenous LH preparation for both cohorts was lutropin alfa (Luveris, Merck Serono). After the COS commencement, the subsequent FSH dosing was adjusted across the COS journey accordingly by the results of the serum hormone levels and transvaginal sonography, as were the type (GnRH agonist, human chorionic gonadotropin, or recombinant human chorionic gonadotropin) and dosage of ovulation trigger medications. All oocytes were retrieved by transvaginal follicular aspiration 34-36h after the injection of ovulation trigger medication. Short GnRH agonist protocol and rFSH preparations were not used in the validation cohort due to center’s preference.

Basal reproductive hormones were measured in serum samples on day 3 of the menstrual cycle. Both AMH and basal reproductive hormones were analyzed using an automated electrochemiluminescence immunoassay (ECLIA; Roche Diagnostics) and recorded within six months before COS commencement. Basal AFC was taken to be the total number of small follicles (diameter: 2–10 mm) on day 2–3 of the menstrual cycle. COS strategies in both the derivation and external validation cohorts were scheduled by each patient’s primary physician (senior attending or above) mainly based on age, body mass index and commonly used ovarian reserve markers.

## Diagnostic criteria of the reproductive disorders in this study

Covariates of reproductive dysfunctions include diagnosis with polycystic ovarian syndrome according to the Rotterdam criteria, and diagnosis with primary ovarian insufficiency (POI) and/or diminished ovarian reserve (DOR). Diagnostic criteria for POI were consistent with the *International Classification of Diseases, tenth Revision, Clinical Modification* (ICD-10-CM): codes E28.3 and E89.4. Women who met all of the following conditions were also considered to have POI (Chinese diagnostic consensus on POI): (1) age <40 years, (2) oligomenorrhea or anovulation for at least four months, (3) basal FSH >25 IU/L on at least two consecutive occasions more than four weeks apart. Women with DOR were identified according to the American Society for Reproductive Medicine (ASRM) committee opinion.

## Artificial intelligence algorithms

*Extreme gradient boosting trees (XGBoost)*. XGBoost is a scalable and high-accuracy implementation of gradient boosting, pushing the limits of computation for tree-based boosting algorithms, built in large part for the purpose of powering artificial intelligence (AI) model performance and computation speed. Gradient boosting is a supervised learning process that combines the predictions of a number of weaker, simpler models to attempt to properly predict a target variable. Regression trees serve as the weak learners when utilizing gradient boosting for regression, and each one of them associates each input data point with a leaf that holds a continuous score. Adding new trees that predict the residuals or mistakes of earlier trees, which are then integrated with earlier trees to get the final prediction, is how the training process is carried out iteratively. It's called gradient boosting because it uses a gradient descent algorithm to minimize the loss when adding new models.

*Support vector machine (SVM) with radial basis functions (RBF)*. To accurately separate two or more distinct classes in a classification problem, a support vector machine finds an ideal separation line, or "hyperplane". By using the SVM technique to train the linearly separable data, the aim is to determine the best hyperplane separation. Formally speaking, the algorithm (SVM) develops a hyperplane of higher dimension space (even if it is not linearly separable), which helps with classification, outlier detection, regression, and other tasks. By using a hyperplane with the greatest distance to the closest training data points, one can effectively separate classes. RBF kernels, one of the most widely used kernels due to its similarity to the Gaussian distribution, are the most generalized form of kernelization for classification of non-linear data.

*Random forest*. Random forest, like its name implies, consists of a large number of individual decision trees that operate as an ensemble. It is a popular model for classification problems. The “forest” it builds is an ensemble of decision trees, usually trained with the “bagging” method with a combination of learning models to improve the overall results.

*Multilayer perceptron*. The multilayer perceptron is a model for a nonlinear mapping between an input vector and an output vector made up of a system of straightforward linked neurons or nodes. The weights and output signals connecting the nodes are a function of the total of the node's inputs, as adjusted by a straightforward nonlinear transfer, or activation, function. The multilayer perceptron may approximate very nonlinear functions by superimposing several basic nonlinear transfer functions.

## Hyperparameter optimization

As a vast number of adjustable configurational parameters of the AI algorithm could greatly influence the performance of derived models, in most cases of this study, the hyperparameters were tuned before each modeling process. A Bayesian optimization method was employed for hyperparameter tuning. By considering past evaluations when choosing which hyperparameters to evaluate next, less iterations are required to achieve the optimal hyperparameter combination, and this approach also brings better generalization performance (metric used: AUC) on the test set. Due to data size and class imbalance, all hyperparameter tuning procedures in this study were based on stratified five-fold cross-validation.

Hyperparameters optimized of each AI algorithm in this study were as follows. *XGBoost*: trees (number of trees, type: integer), tree depth (type: integer), learning rate (type: double), mtry (randomly selected predictors, type: integer), min_n (minimal node size, type: integer), loss reduction (minimum loos reduction, type: double), sample size (proportion observations sampled, type: double); *SVM-RBF*: margin (the epsilon in the SVM insensitive loss function, type: double), cost (cost of predicting a sample within or on the wrong side of the margin, type: double), Rbf-sigma (radial basis function sigma, type: double); *Random forest*: trees (number of trees, type: integer), mtry (randomly selected predictors, type: integer), min_n (minimal node size, type: integer); *Multilayer perceptron*: penalty (amount of regularization, type: double); *Generalized linear model (logistic regression with lasso)*: penalty (amount of regularization, type: double); *Generalized linear model (logistic regression with ridge)*: penalty (amount of regularization, type: double).

## Feature selection and explainable predictions

A strategy-model-determined scheme was used to reduce the complexity of the modeling process. That was, the top important baseline features and algorithm for developing four submodels were determined by evaluating the full-variable strategy models (incorporating all 55 features) developed by six representative AI algorithms.

Shapley additive explanations (SHAP), based on “Shapley values” originally inspired by the cooperative games, was applied for obtaining feature importance and subsequent feature selection in this study. SHAP is theoretically optimal and has been verified as the only method maintaining two important properties simultaneously: local accuracy (the sum of all feature attributions plus the mean prediction should equal the output of the model) and global consistency (the importance estimate assigned to a particular feature should not decrease when the model is updated). Briefly, a Shapley value is calculated as the average contribution of a feature value to the prediction in the context of its interaction with other features in different coalitions. On this basis, a high mean absolute SHAP value (from the overall data or a subsample) of a particular feature suggests a large impact on the outcome and thus the variable is important. Lundberg and Lee who proposed the SHAP method also developed a fast solution to calculate exact SHAP values for tree-based models which was adopted in this study. In this present study, COS protocol, FSH starting dose, using rFSH (or uFSH) and exogenous LH supplementation were always integrated to develop strategy models as they were all vital OS therapeutic components in clinical practice. Instead of selecting a fixed number (like 15 or 20), top important features to be included in final submodels were selected in a model-tailored SHAP ranking manner—determined by the decline speed of mean absolute SHAP values as the ranking number of features increased in the best-performing full strategy models.

Feature contributions were globally interpreted and visualized by SHAP summary plots where each point was a mean absolute Shapley value for a specific feature and instance. To harmonize various distributions and scales across different features in a SHAP summary plot, several preprocessing steps were performed to continuous variables, including the Yeo-Johnson normalization transformation, elimination of outliers, and scaling to a same predefined range. Local explanations of the submodels were also investigated to show the patient-specific impact of key risk factors on POR and HOR predictions, using three representative patients assessed by the reproductive experts in advance: (1) LORRM predicting a patient with an expected high POR risk, (2) HORRM predicting a patient with an expected high HOR risk, and (3) both LORRM and HORRM predicting a patient with an expected normal ovarian response. In the local explanations of the submodels on the patient-specific level, the average contributions of features were computed under 100 different feature orderings to mitigate the influence of the features’ order.

## Design of superiority, sensitivity, and subgroup analyses

Forty comparisons were conducted to determine whether the submodels outperformed the models using widely used ovarian reserve markers (ORMs) (for comparing strategy submodels, the four schedulable COS components were added): (1) with only age; (2) with only FSH; (3) with only AFC; (4) with only AMH; (5) with age and FSH; (6) with age and AFC; (7) with age and AMH; (8) with age, AMH and FSH; (9) with age, AMH and AFC; (10) with age, AMH, FSH and AFC.

Sixteen tests in total were performed for sensitivity and subgroup analysis. First, we evaluated all submodels in the external validation cohorts where AFC and AMH were unavailable (i.e., setting AFC and AMH values to missing), respectively. Second, we performed two age-related tests: evaluating the performance of LORRM and LORSM on younger women (age < 35y) who would be less likely to suffer from POR; evaluating the performance of HORRM and HORSM on older women (age ≥35y) who would less likely develop HOR. Last, we performed the ORM-related tests: assessing the performance of LORRM and LORSM on individuals with good ovarian reserve (AMH >1.1ng/mL or AFC >5) and assessing the performance of HORRM and HORSM on individuals at low risk of HOR (AMH ≤3.4ng/mL or AFC ≤24).

## Deployment of individualized COS strategies using the AI-based system

There are many different ways for implementation of the AI-based system. Here is an example of our web application. The implementation of the risk prediction model is simple, i.e., providing the input interface for top important baseline features and submitting the input values to LORRM and HORRM for prediction. As for the implementation of how to deploy COS strategies using the strategy submodels, we performed the following steps:

1. Provide the input interface of top important baseline features.

2. Submit the 83 predefined COS therapeutic decision combinations with the input baseline features to LORSM and HORSM, respectively, to make 83 predictions of abnormal ovarian responses. These 83 COS treatment decision combinations were set up as follows: COS protocol (4 choices: long GnRH agonist, ultra-long GnRH agonist, Short GnRH agonist, GnRH antagonist) × FSH starting dose (5 choices: ≤ 100 IU, 150 IU, 200 IU, 225 IU, ≥ 300 IU) × Using rFSH or uFSH (2 choices: rFSH, uFSH) × LH supplementation (2 choices: yes, no) + COS protocol (3 choices: PPOS, mild or natural cycle, other) × FSH starting dose (1 choice: non-GnRH) × Using rFSH or uFSH (1 choice: non-GnRH) × LH supplementation (1 choice: non-GnRH) = 4 × 5 × 2 × 2 + 3 × 1 × 1 = 83.

3. Rank the 83 predicted non-POR and 83 non-HOR probabilities from largest to smallest (i.e., rank the predicted POR and HOR risks from smallest to largest).

4. Summary the results of step 3 and present the corresponding four COS treatment decisions (full scanning function). The proposed strategies should be sorted in descending order according to the predicted non-POR/non-HOR risks. We recommend only top N strategies (e.g., N = 5) can be taken into consideration, because the confidence intervals of non-POR/HOR risks of the proposed strategies ranked behind may cross downwards to the threshold.

5. Provide the input interface for the 4 COS treatment decisions, then submit the input information together with the input baseline characteristics to LORSM and HORSM for prediction and display the prediction results (specific testing function).

Since different hospitals or reproductive centers have different preferences for COS treatments, we strongly recommend that the selection of proposed COS strategies (combinations of four COS treatment components) should be made based on actual conditions. In addition, some COS strategies may be used in a relatively small proportion of the population, resulting in insufficient confidence for these predicted COS strategies. Therefore, we emphasize that the predictions of the strategy submodels are only for reference purposes before they are adequately validated.
